# Modeling cross‐talk of RNA modification enzymes reveals tumor microenvironment‐associated clinical significance and immunotherapy prediction in hepatobiliary malignancy

**DOI:** 10.1002/mco2.256

**Published:** 2023-04-18

**Authors:** Feng Qi, Jia Li, Zhuoran Qi, Bin Zhou, Biwei Yang, Jun Zhang, Wenxing Qin

**Affiliations:** ^1^ Phase I Clinical Trial Center, Department of Oncology, Shanghai Medical College Fudan University Shanghai Cancer Center Fudan University Shanghai China; ^2^ Department of Oncology Ruijin Hospital Shanghai Jiao Tong University School of Medicine Shanghai China; ^3^ Liver Cancer Institute Zhongshan Hospital Fudan University Shanghai China; ^4^ Department of Hepatic Surgery VI Eastern Hepatobiliary Surgery Hospital Second Military Medical University Shanghai China

**Keywords:** hepatobiliary malignancy, immunotherapy, RH_Score, RNA modification, tumor microenvironment

## Abstract

RNA modification includes four main types, N6‐methyladenosine, N1‐methyladenosine, alternative polyadenylation (APA), and adenosine‐to‐inosine (A‐to‐I) RNA editing, involving 41 enzymes that serve as “writers”, “readers” and “erasers”. By collecting RNA modifying enzyme information in 1759 hepatobiliary malignancy (HBM) samples from 11 datasets, an RNA modification HBM Score (RH_score) was established based on unsupervised cluster analysis of RNA modification‐associated differentially expressed genes (DEGs). We identified the imbalanced expression of 41 RNA modification enzymes in HBM, which was scientifically categorized into two groups: RH_Score high and RH_Score low. A high RH_Score was associated with a worse prognosis and more immature immune cells in the tumor microenvironment (TME), while a low RH_Score was associated with a better prognosis and more mature immune cells in the TME. Further analysis using single‐cell databases showed that the high RH_Score was immune exhaustion in the TME. RH_Score was involved in transcriptional regulation and post‐transcriptional events in HBM. Additionally, resistant and sensitive drugs were selected based on RNA modification, and anti‐PD‐L1 therapy responded better with low RH_Score. In conclusion, our study comprehensively analyzes RNA modification in HBM, which induces TME changes and transcriptional and posttranscriptional events, implying potential guiding significance in prognosis prediction and treatment options.

## Graphical abstract

Based on RNA modification‐associated differentially expressed genes from 1659 hepatobiliary malignancy (HBM) samples from 10 datasets, an RNA modification HBM Score (RH_score) is scientifically established and tested in 100 hepatocellular carcinoma clinical samples, which demonstrates excellent efficacy in terms of prognosis and targeted treatment. A high RH_Score means a worse prognosis and more immature immune cells, while a low RH_Score has the opposite result.

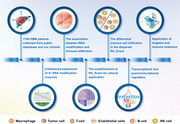

## 1 INTRODUCTION

Hepatobiliary malignancy (HBM) is a leading cause of cancer‐related death in the world,^1^ consisting of hepatocellular carcinoma (HCC), cholangiocarcinoma, and the mixed pathological subtype.^2^ For many years, researchers have focused on uncovering the initiation and development of HBM and found that the initiation of HBM is the result of the accumulation of mutations.^3^ Meanwhile, epigenetic changes in cancer‐related genes may also exert a critical function on the etiology of HBM.^4^ Epigenetics is the study to explain how the genome is interpreted through the cell to generate a phenotype.^5^ In recent years, more and more studies have found that RNA modification is a critical mechanism of epigenetic regulation and exerts a key effect on the progression of cancers.^6^

Many kinds of RNA modifications have been well documented.^7^ In addition to canonical A, C, G, and U residues, modified nucleotides have been detected in many cellular RNAs.^7^ N6‐methyladenosine (m6A) is a type of RNA modification, and other modification methods, such as N1‐methyladenosine (m1A), alternative polyadenylation (APA), and adenosine‐to‐inosine (A‐to‐I) RNA editing, affect RNA splicing, stabilization, transportation, nucleation, positioning, translation, and other processes.^8^, ^9^ Through the influence of RNA, RNA modification assists, strengthens, and even reverses the expression of DNA, which leads to changes in the function of cells and the microenvironment surrounding cells. Focusing on the details of RNA modification, “writers”, “erasers” and “readers” serve as coregulators. Recent studies have found the important functions of coregulators, although how these coregulators work together and whether cross‐talk effects exist in HBM still need to be resolved.

The tumor microenvironment (TME) is crucial to the onset and progression of tumorigenesis,^10^ and it consists of a complex network of structures, including multipotent stromal cells or mesenchymal stem cells, fibroblasts, blood vessels, endothelial cell precursors, immune cells, and various cytokines.^11^ Many studies have found that RNA modification is highly correlated with TME and related immune cells. m6A modification patterns in individual tumors could help to predict tumor inflammatory stage, subtypes, TME stromal activity, genetic variation, and patient prognosis.^12^ In patients with colon cancer, m1A modification is associated with the proliferation of CD8+ T effectors, in addition to high microsatellite instability, neoantigen burden, and PD‐L1 expression, thus demonstrating prolonged survival and better response to anti‐PD‐L1 immunotherapy.^13^ When the immune cells or other cell types present in the TME differed, the 3′UTR patterns were different.^14^ However, these studies were confined to a few kinds of RNA modification types and thus could not explain the complex regulatory effects of RNA modifications. Therefore, comprehensively exploring the regulatory network of various RNA modifications, especially influencing the TME, will help us to better understand the development of immunotherapy strategies for HBM.

In this study, we identified 41 RNA modification enzymes to identify the association between the TME and the unbalanced expression of these enzymes from the Gene Expression Omnibus (GEO) and The Cancer Genome Atlas (TCGA). Furthermore, we designed an RH score model to predict the clinical prognosis, response to molecular targeted drugs and immunotherapy, and transcriptional and posttranscriptional events based on RNA modification‐regulated DEGs, thereby providing a novel panel of next‐generation sequencing for clinical translation.

## 2 RESULTS

### 2.1 Genetic landscape of four types of RNA modification regulators in HBM

In this study, we selected 41 RNA modification regulators (Table S1) to perform our research according to publicly available data,^15^, ^16^ including seven m6A modification “writers”, 4 m6A/m1A modification “erasers”, 11 m6A modification “readers”, three A‐I modification “writers”, four m1A modification “writers”, and 12 APA modification “writers”.

**FIGURE 1:**
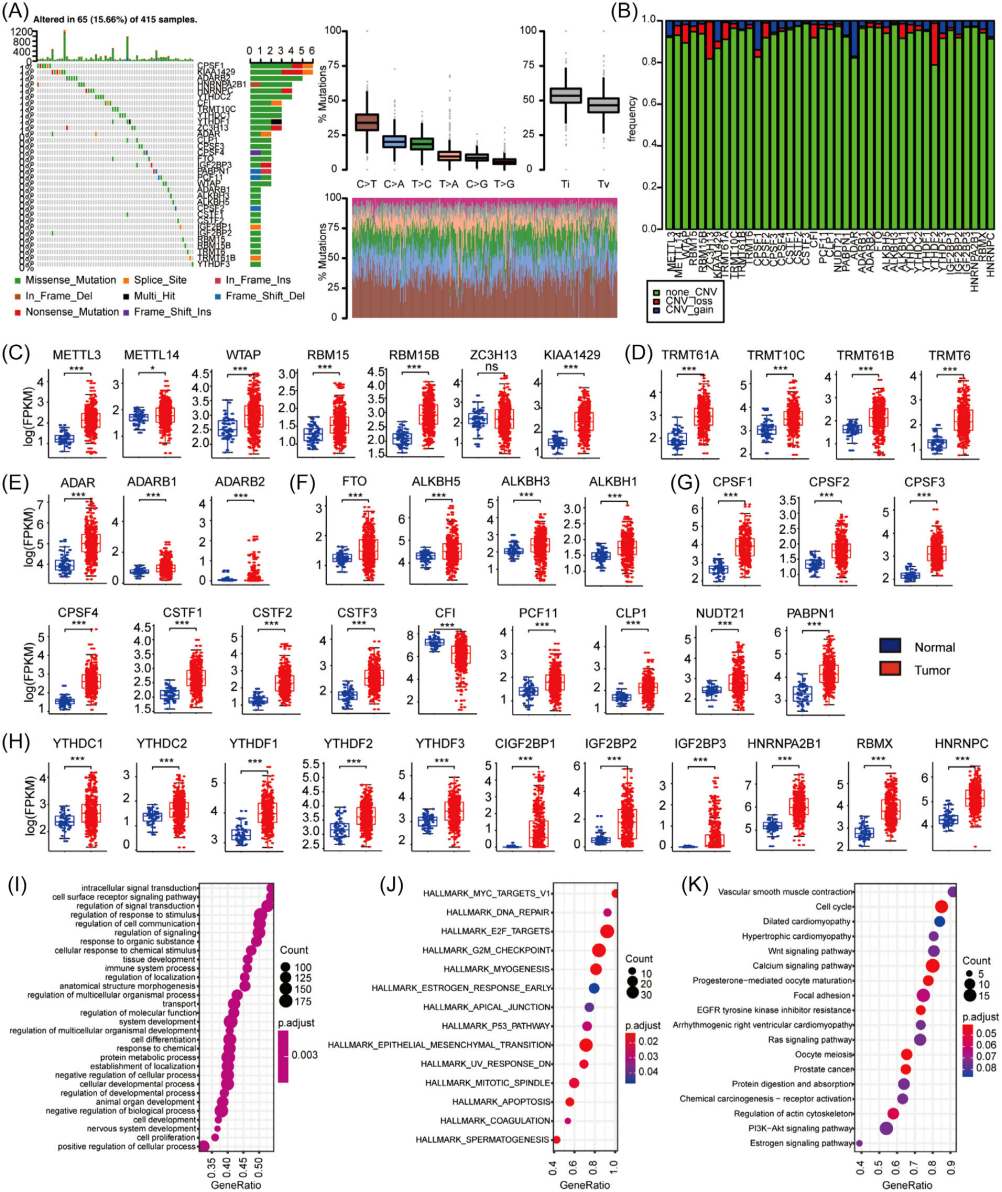
Genetic and transcriptional changes related to RNA modification regulators in hepatobiliary malignancy (HBM). (A) Mutation frequencies related to 41 RNA modification regulators in 415 HBM patients from the Cancer Genome Atlas (TCGA) cohort. Patients were represented using each column. Tumor mutation burden (TMB) was shown in the upper bar chart; the frequency of mutations in each regulator was represented by the left number. the proportion of variant types was shown in the right bar graph. The fraction of conversions was shown in the stacked bar graph. (B) Bar graphs representing copy number variation (CNV) gain frequency (blue), loss frequency (red), and non_CNV frequency (green) of RNA modification regulators. The alteration frequency was represented by the height of each bar. (C–H) Box plots representing RNA modification regulators’ expression distribution in paired normal (blue) and HBM (red) tissues, N6‐methyladenosine writer (C), N1‐methyladenosine (m1A) writer (D), A‐I writer (E), m1A/m6A eraser (F), alternative polyadenylation (APA) writer (G), m6A reader (H); The boxes indicate the median ± 1 quartile. (I–K) Bubble charts displaying the different enrichment in the characteristics of genome ontology (GO) (I), genome set variation analysis (GSVA) (J), and Kyoto Encyclopedia of Genes and Genomes (KEGG) (K) analysis between the regulator mutation group and nonmutant group in the TCGA cohort. (Level of significance: ***, *p* < 0.001; *, *p* < 0.05; ns, *p* > 0.05).

First, the prevalence of nonsilent somatic mutations in 41 regulators was assessed in HBM. Consistent with a previous study,^17^ our results showed that the HBM mutation frequency of RNA modification regulators indeed existed in TCGA. Mutations in RNA modification regulators occurred only in 65 (15.66%) out of the 415 HBM samples (Figure 1A). In detail, the mutation frequency of CPSF1 and KIAA1429 was the highest (1.45%), followed by ADARB2, HNRNPA2B1, HNRNPC and YTHDC2, while METTL3, METTL14, TRMT61A, CSTF3, NUDT21, ALKBH1, YTHDF2, and EIF3 did not show any mutations in HBM. Next, the somatic copy number variation (CNV) and mRNA expression of these regulators were examined (Figure 1B). Because of the existence of these regulators’ copy number alterations, their mRNA expression showed a mostly upward trend (Figure 1C–H). Interestingly, we found that many regulators with genetic mutations or CNV gains were significantly increased in HBM. These results prompted us to conclude that genetic mutation or CNV may be very important to the mRNA expression of these regulators. However, some regulators increasing in HBM also showed a high frequency of CNV loss, such as CPSF1, CPSF2, CPSF3, CPSF4, and RBM15B (Figures S1 and S2). Tumor formation is a complicated process, and gene mutations or CNVs cannot fully elucidate the differences in the expression of RNA modification regulators but rather can only partially reveal the variations. Other characteristics, including DNA methylation and transcription factors, may also exert a very important function in HBM.^18^

Next, genome ontology (GO) analysis, genome set variation analysis (GSVA), and Kyoto Encyclopedia of Genes and Genomes (KEGG) enrichment analysis were performed to compare the mutant and non‐mutant groups of regulatory genes in the TCGA cohort (Figure 1I–K). We defined at least one gene mutation of 41 RNA modification regulators in the samples as a mutant group. And the differences in the genetic screening process were referred to in the contents of The R LIMMA in the attached file. the package was used to analyze significantly different genes based on total mRNA expression values: log2 transformation fragments per kilobase million (FPKM) of tumor samples from different clusters. FDR < 0.05 and |log2FC | > 1 were set as the thresholds for filter differences. The results of the GO analysis showed that more signal transduction pathways and immune system processes were enriched in the mutation group. GSVA and KEGG analysis found that more cell cycle‐ and autophagy‐related signaling pathways were activated, such as Myc targets, DNA repair, G2 M checkpoint, E2F targets, P53 signaling pathway, epithelial‐mesenchymal transition, cell cycle, and Wnt/Ras/PI3K‐Akt signaling pathway. Studies have shown that DNA damage response pathways promote many key steps in the carcinogenesis process. The production of one or more mutations may directly or indirectly lead to oncogene activation, resulting in replication and/or oxidative stress. In addition, the activation of the Wnt/Ras/PI3K‐Akt pathway is also important for the formation of HBM. These results suggest that mutations in RNA modification regulators may lead to functional changes that affect HBM progression. Taken together, the genetic landscape and expression of RNA modification regulators between normal and HBM groups uncovered high heterogeneity, suggesting that RNA modification imbalance exerted an indispensable function on HBM development.

### 2.2 Remarkable characteristics of the metabolism and immune infiltration based on RNA modification regulators

**FIGURE 2:**
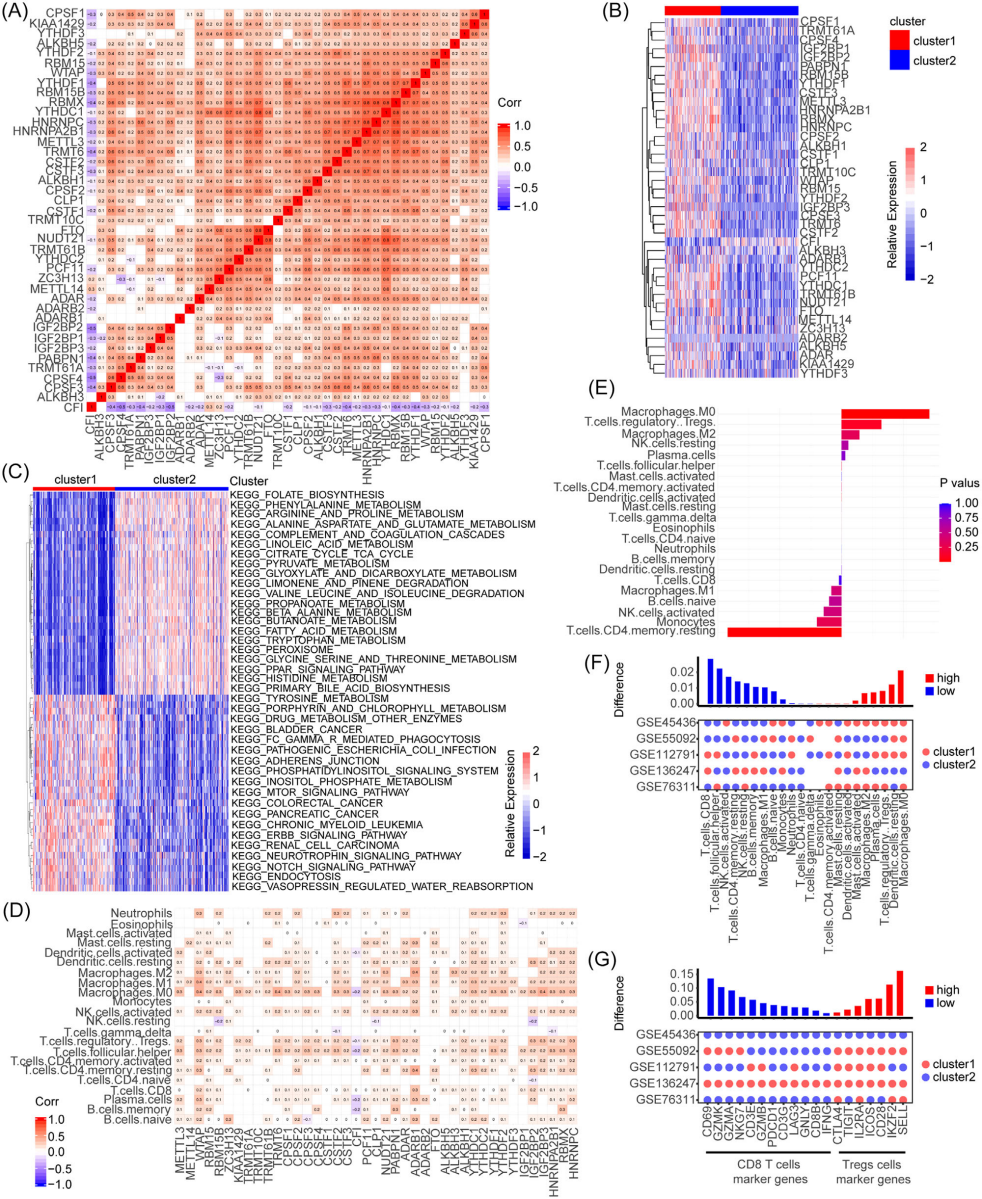
Cluster patterns of RNA modification and biological features of metabolism and immune infiltration in each cluster. (A) Heatmap displaying the correlation among 41 RNA modification regulators in hepatobiliary malignancy (HBM) using Spearman correlation analysis. Corr: correlation; Color bar: positive (red); negative (blue); Number: degree of correlation. (B) Unsupervised clustering of 41 regulators. The clusters of HBMs were used as sample annotations (top bar color label). Red (high expression); blue (low expression). Color bar: positive (red); negative (blue); Number: degree of correlation. (C) Heatmap displaying the Kyoto Encyclopedia of Genes and Genomes (KEGG) enrichment analysis from distinct RNA modification clusters. The clusters of HBM used as sample annotations. Red (activated pathways); blue (inhibited pathways). Color bar: positive (red); negative (blue); Number: degree of correlation. (D) Heatmap displaying the correlation between RNA modification regulators and immune cells in HBM using Spearman correlation analysis. Positive (red); negative (blue). (E) The difference in immune cell infiltration in the TME between RNA‐modified cluster1 and RNA‐modified cluster2 was assessed by the CIBERSORT algorithm based on the GSE76311 dataset. Difference > 0 indicates that the immune cells were enriched in RNA‐modified cluster1, and the column color indicates that the difference was statistically significant. (F) Difference of immune cell infiltration in RNA‐modified patterns in the five datasets. The difference between cluster1 and cluster2 was shown in the upper bar graph. The number of cluster1 or cluster2 in each of the distinct GEO datasets was represented by the color of the bubble below the graph. Differences (high, red) indicate that the immune cells’ infiltration was higher in RNA‐modified cluster1; differences (low, blue) indicate that the immune cells’ infiltration was higher in the RNA‐modified cluster. (G) Expression of CD8+ T cell and Treg markers between RNA‐modified patterns in five datasets. The difference between cluster1 and cluster2 was shown in the upper bar graph. The number of cluster1 or cluster2 in each of the distinct GEO datasets was represented by the color of the bubble below the graph. Differences > 0 (red) indicate that the expression of Treg markers was higher in RNA‐modified cluster1; differences < 0 (blue) indicate that the expression of CD8+ T cell markers was higher in RNA‐modified cluster2.

To comprehensively understand the expression patterns of RNA modification regulators in HBM, a total of 424 samples from TCGA with clinical data were chosen for further study (Table S2). A univariate Cox regression analysis indicated that 28 out of 41 RNA modification regulators were associated with the prognosis of HBM (Figure S3A). A pairwise correlation analysis showed that the expression of 41 regulators in HBM exerted much more frequent positive correlations than negative ones (Figure 2A). Significant correlations existed not only between the same class of RNA modifications but also between the disparate types of modifications. It is worth noting that only the expression of CFI was negatively correlated with other regulators (Figure 2A). Therefore, we speculated that the crosstalk among RNA modification regulators may have important complementary effects on HBM.

Then, consensus clustering was performed according to the expression characteristics of the 41 regulators to divide patients into diverse patterns: 169 HBM patients were identified in cluster1 and 234 were classified in cluster2 (Figure 2B). A Kaplan‐Meier analysis showed that the survival of patients in cluster1 was significantly lower than that of patients in cluster2. (Figure S3B; *p* < 0.001). Next, we performed a KEGG enrichment analysis to evaluate the biological significance of these two clusters (Table S3). Cluster1 was enriched in cancer‐related pathways, including the mTOR signaling pathway, Notch signaling pathway, and cell cycle, whereas cluster2 was enriched in pathways related to metabolism‐related pathways, consisting of amino acid metabolism, lipid metabolism, glucose metabolism, and drug metabolism (Figure 2C). These results suggested that RNA modification regulators were involved in the development and metabolic processes of HBM.

Numerous studies have reported that the effect of RNA modification on tumors cannot be separated from the intervention of immune cell infiltration.^19^ Therefore, the function of RNA modification regulators in the TME was further studied. Pairwise correlation analysis from TCGA showed that strong correlations existed between the expression of 41 regulators and immune cells in HBM (Table S4). Consistent with previous findings, positive correlations with immune cells were rather frequent, except for CFI (Figure 2D). To gain a better understanding of the relationship between immune cell infiltration and RNA modification, 153 HBM samples from GEO datasets (GSE76311) were selected for further analysis (Table S2) with the CIBERSORT method.^20^ The expressions of 41 regulators in the dataset are shown in Figure S4C. The differences in TME cell infiltration in different clusters were analyzed. In cluster1, more immunosuppressive cells infiltrated, such as T regulatory cells (Tregs), M2 macrophages, resting NK cells, and plasma cells. However, in cluster2, more immune‐promoting cells, such as CD8+ T cells, M1 macrophages, B cells, activated NK cells, monocytes, and resting CD4+ memory T cells, were enriched (Figure 2E). To verify the accuracy of the conclusion, we added 4 other GEO datasets (GSE136247, GSE112791, GSE55092, and GSE45436) (Figure S4), and the cumulative number of patients increased to 517. Similar results were obtained with the increased proportion of Tregs in cluster1 and CD8+ T cells in cluster2 (Figure 2F). Based on these findings, we further explored the expression of related markers, including membrane proteins, stimulating genes, and subtype biomarkers reported before.^21^ The results indicated that the Treg marker genes CTLA4, TIGIT, IL2RA, ICOS, CD28, IKZF2 and SELL were significantly upregulated in cluster1, while the CD8+ T cell marker genes CD69, GZMK, GZMA, NKG7, CD3E, GZMB, PDCD1, CD3G, LAG3, GNLY, CD8B, and IFNG were significantly upregulated in cluster2 (Figure 2G). These results suggested that the degree of infiltration of specific immune cell types was influenced by specific RNA modification patterns.

### 2.3 Establishment of RNA modification molecular signatures and RH_Score with clinical characteristics in HBM

**FIGURE 3:**
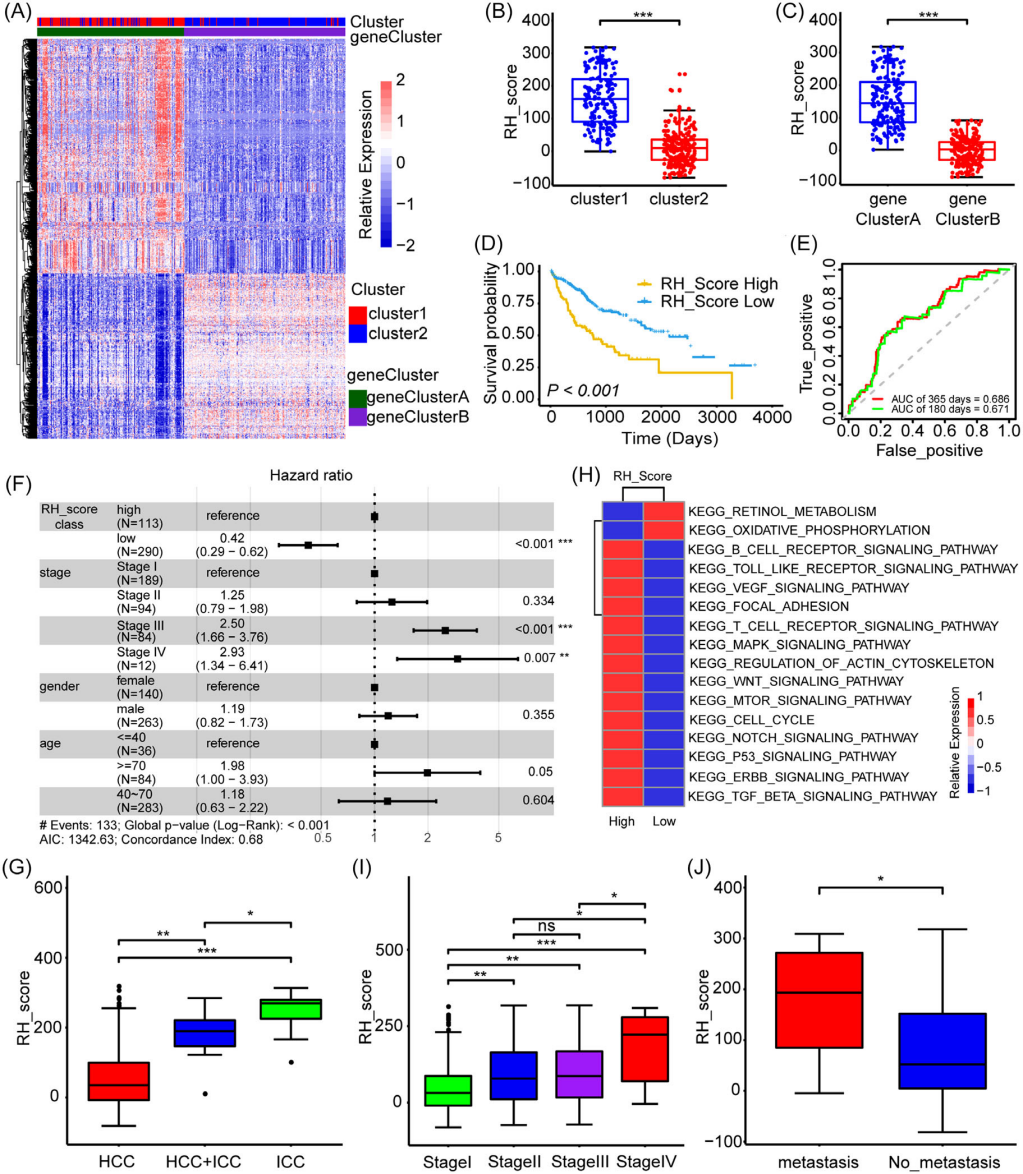
Establishment of RNA modification signatures and RH_Score with clinical characteristics in hepatobiliary malignancy (HBM). (A) Unsupervised clustering of the RNA‐modified phenotype‐related genes. The clusters of HBM were used as sample annotations. Red (high expression); blue (low expression) Color bar: positive (red); negative (blue); Number: degree of correlation. (B, C) Differences in RH score between RNA‐modified clusters (B) and gene clusters (C) in The Cancer Genome Atlas (TCGA) cohort. Wilcoxon test was used, and *p* < 0.05 was considered statistically significant. (D) Kaplan‐Meier curve displaying overall survival in RH_Score‐high (yellow) and RH_Score‐low (blue) TCGA cohort. The bottom of the chart shows the grouping of HBM. The difference was statistically significant when *p* < 0.05 in the log‐rank test. (F) Predictive value of the RH score in patients from the TCGA cohort (area under the curve [AUC]: 0.671 and 0.686; 180‐ and 365‐day overall survival). (F) Multivariate Cox regression model analysis, consisting of the RH score, patient age, sex, stage, and patient outcomes in TCGA. The length of the horizontal line represents the 95% confidence interval (CI) of each group. The vertical dotted line represents the risk ratio (HR) of all patients in the forest plot. (G) Differences in RH score among pathological subtypes of HBM from the TCGA cohort. (H) Heatmap displaying the differences in pathways enrichment between the high RH_Score and low RH_Score groups from the TCGA cohort. Red (high enrichment score); blue (low enrichment score). The number beside the color bar is correlation coefficient. (I) Differences of RH score in diverse TNM stages of HBM from TCGA. (J) Differences of RH score between the metastasis (red) and nonmetastasis (blue) groups in TCGA. Wilcoxon test was applied. The boxes show the median ±1 quartile. (Level of significance: ***, *p* < 0.001; **, *p* < 0.01; *, *p* < 0.05; ns, *p* > 0.05).

Furthermore, 924 DEGs associated with RNA phenotypes based on the TCGA database were identified, and related enrichment analysis was performed to clarify the functional role of the two clusters mentioned above. GO analysis showed that some biological processes, especially those related to the protein activation cascade, catabolic progress, acute inflammatory response, and some metabolic processes (Figure S5A and Table S5), were enriched. KEGG analysis showed that metabolism‐related signaling pathways were enriched, such as amino acid metabolism, lipid metabolism, glucose metabolism, and drug metabolism (Figure S5B and Table S6). Next, we validated these differential regulations through unsupervised cluster analysis of these 924 genes, classifying the patients with two subtypes: geneClusterA and geneClusterB (Figure 3A and Table S7), which is similar to the clustering of RNA modification patterns. For example, geneClusterA included 192 HBM patients, 149 out of which belonged to cluster1, while geneClusterB included 211 patients, 192 out of which belonged to cluster2. The prognosis of patients in group geneCluster A was worse than that in group geneClusterB (Figure S5C). To comprehensively understand the heterogeneity and complexity of RNA modifications, a DEG‐based score model based on 524 phenotype‐related genes was set up to quantify the RNA modification patterns in HBM, which was termed the RH_Score (RNA Modification Regulator Score in HBM; see Methods). These 524 genes were chosen from the 924 DEGs mentioned above according to univariate Cox regression analysis (*p* < 0.05). We used this model to calculate the scores of various classifications, finding that the score in cluster1 was higher than that in cluster2 (Figure 3B), and the score in geneClusterA was higher than that in geneClusterB (Figure 3C). Next, the immune cell infiltration was compared to evaluate the function of the RH score on the TME. We found that Tregs, T follicular helper cells, and NK resting cells were enriched in the RH_Score high group, and CD8+ T cells and NK‐activated cells were enriched in the RH_Score low group (Figure S5D). Then, an overlap analysis based on the Wayne diagram and the histogram of frequency distribution was performed to study these three different classifiers in detail. Of the 113 samples with a high RH_Score, 108 (95.58%) overlapped with samples in cluster1, and all (100%) overlapped with samples in geneClusterA; of the 290 samples with a low RH_Score, 229 (79.0%) overlapped with samples in cluster2, and 211 (72.8%) overlapped with samples in geneClusterB (Figure S5E–H). The results showed that the three classification methods have excellent consistency and that the RH_Score could reveal the RNA modification patterns of HBM patients.

Then, the clinical value of the RH score model was assessed. We determined the cut‐off value by the survminer package to classify patients into two groups (high RH_Score and low RH_Score), discovering that patients with high RH_Score had poorer survival (Figure 3D). The AUCs of the time based on ROC curves for the RH score were 0.671 and 0.686 for 180‐ and 365‐day overall survival (Figure 3E). Additionally, multivariate Cox regression analysis showed that the RH score may be an excellent independent prognostic biomarker in HBM patients (Figure 3F). These results suggested that the RH score could forecast the prognosis of HBM patients. In addition, we used 100 HCC samples from Fudan University Shanghai Cancer Center to validate the application of RH_Score. Similarly, we constructed the RH_Score model with high RH_Score and low RH_Score groups. HCC patients with high RH_Score had poorer survival. The AUCs of the time based on ROC curves for the RH score were 0.963 and 0.926 for 180‐ and 365‐day overall survival. Additionally, multivariate Cox regression analysis showed that the RH score may be an excellent independent prognostic biomarker in a validated cohort (Figure S7 and Table S8).

In this study, HBM included three pathological types of liver cancer: HCC, intrahepatic cholangiocarcinoma (ICC), and mixed type (HCC+ICC). Among the three types of liver cancer, ICC is reported to have the highest malignant degree and the worst prognosis. To understand the relationship between the RH score and HBM pathological subtypes, the RH score of different HBM subtypes in the TCGA cohort was analyzed. We found that different HBM subtypes showed a significant difference in RH_Score, the RH_Score of the ICC group was the highest (Figure 3G). Additionally, the distribution of HBM subtypes was significantly diverse in the high RH_Score and low RH_Score groups. The ICC was more common in the high RH_Score group, while HCC was more common in the low RH_Score group (Figure S6A). We further analyzed the HBM‐related pathways characteristic of the diverse HBM subtypes,^22^ showing that the high RH_Score group presented more tumorigenic characteristics, such as the MAPK signaling pathway, WNT signaling pathway, mTOR signaling pathway, Notch signaling pathway, P53 signaling pathway, cell cycle, and TGF‐β signaling pathway, similar to previous findings (Figures 2C and 3H). Notably, these pathways obtained the highest enrichment in ICC (Figure S6B), explaining its poor prognosis. We then demonstrated that the RH_Score was various across tumor stages and was higher in more advanced and metastatic HBM (Figure 3I,J), suggesting that the RH_Score was involved in tumor progression. Consistently, the ICC subtype was mostly in the stage IV group, while the HCC subtype was predominant in stage I/II/III groups (Figure S6C). These results suggest that the RH_Score is closely related to HBM pathological types, and a high RH_ score may be involved in a poor prognosis by activating the MAPK signaling pathway, WNT signaling pathway, mTOR signaling pathway, Notch signaling pathway, P53 signaling pathway, cell cycle and TGF‐β signaling pathway and other signaling pathways mediating tumor progression.

### 2.4 Distinctive immune ecosystems in RNA modification molecular signatures

**FIGURE 4:**
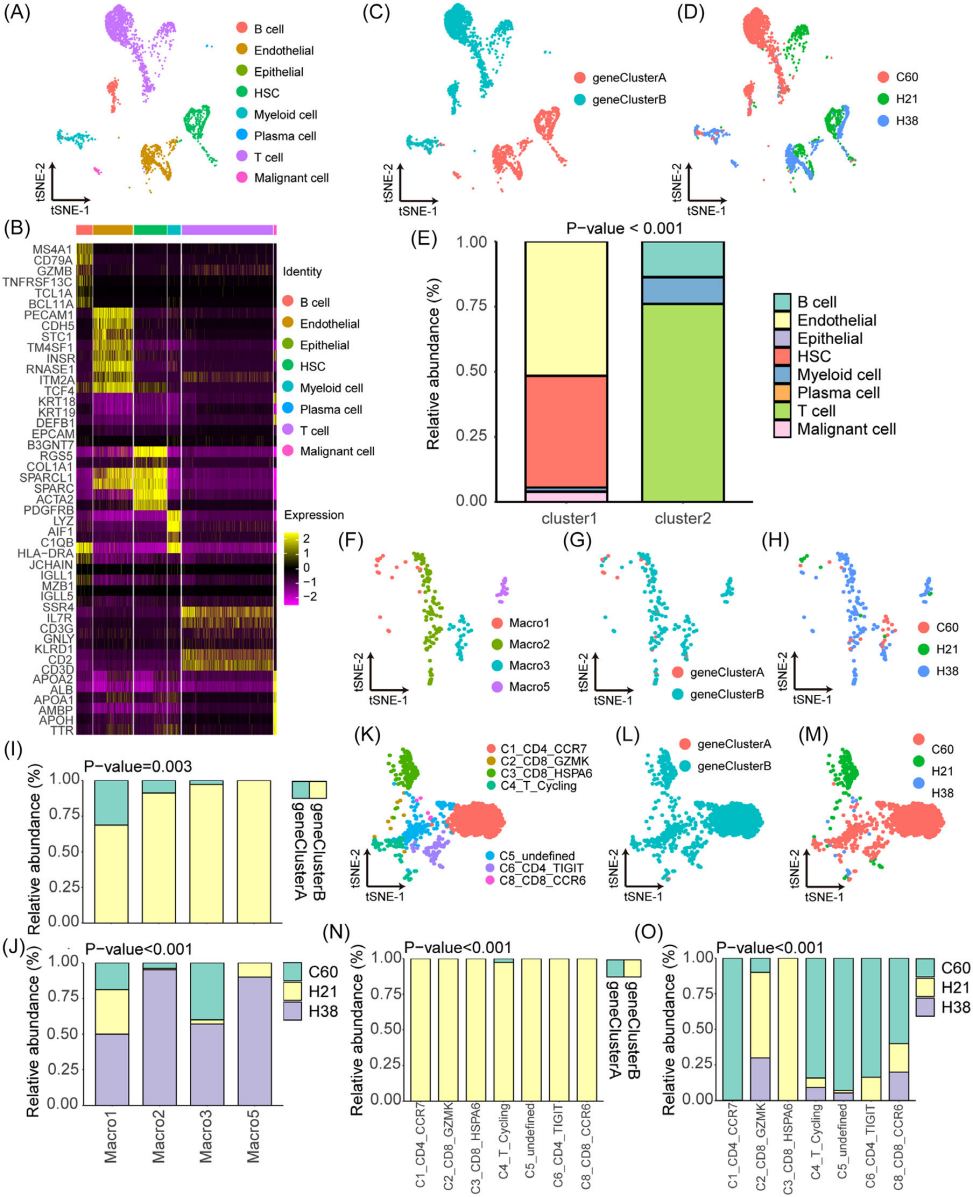
Immune ecosystem of RNA modification molecular signatures. (A) T‐distributed stochastic neighbor embedding (t‐SNE) plot representing the annotation and color coding for cell types in patients without anti‐PD‐L1 treatment. (B) Heatmap displaying the expression of marker genes in the indicated cell types. The color of the top bar marks the cluster corresponding to specific cell types. (C) t‐SNE plot displaying the origin of cells by color and geneCluster (right panel). (D) t‐SNE plot displaying the origin of cells by color and patient origin (right panel). (E) Histogram indicating the proportion of cells in geneClusterA and geneClusterB. (F–H) T‐distributed stochastic neighbor embedding (t‐SNE) plot, showing the annotation and color codes for macrophage types in the hepatobiliary malignancy (HBM) ecosystem (F), showing cell origins by color (G), and showing cell origins by color (H). (I) Histogram indicating the proportion of cells in geneClusterA and geneClusterB. (J) Histogram indicating the proportion of cells in tumor tissue of each analyzed patient. (K–M) T‐distributed stochastic neighbor embedding (t‐SNE) plot, showing the annotation and color codes for T cell types in the HBM ecosystem (K), showing cell origins by color (L), and showing cell origins by color (M). (N) Histogram indicating the proportion of cells in geneClusterA and geneClusterB. (O) Histogram indicating the proportion of cells in tumor tissue of each analyzed patient. *p* < 0.05 was considered statistically significant.

To comprehensively elaborate the immune environment in RNA modification molecular signatures, we performed a global niche atlas with cell classification and marker gene identification using a single‐cell sequencing dataset (GSE125449) without anti‐PD‐L1 treatment from the NCBI Public Data Platform (Tables S9 and 10). Cells with retained cell gene numbers and UMI numbers within ± 2 times the mean standard deviation and mitochondrial gene proportion less than 10% were considered high‐quality cells for downstream analysis. We identified and visualized 8 clusters using the T‐distributed stochastic neighbor embedding (t‐SNE) method (Figure 4A,B; STAR methods), including B cells (MS4A1, CD70A, TNFRSF13C, and BCL11A), endothelial cells (PECAM1 and CDH5), epithelial cells (KRT18, KRT19, DEFB1, and EPCAM), hepatic stellate cells (HSC; RGS5, ACTA2, and PDGFRB), myeloid cells (LYZ, AIF1, and C1QB), plasma cells (JCGAIN, MZB1, and SSR4), T cells (IL7R, CD3G, CD2, and CD3G) and malignant cells (APOA2, ALB, APOA1, AMBP, APOH, and TTR). Based on RNA modification molecular signatures, we found distinctive cell component clusters. In geneClusterA, nonimmune cell subtypes (only endothelial cells, epithelial cells, HSCs, and malignant cells) were enriched, while more immune cell subtypes (B cells, myeloid cells, plasma cells, and T cells) were enriched in geneClusterB (Figure 4C,E). These results suggested that the good prognosis of patients in geneClusterB may be due to the presence of more immune cells in the TME to help devastate tumor cells. In addition, we performed cell classification depending on the three patients and found that ICC (C60) patients enriched more T cells than HCC patients (H21 and H38) (Figure 4D). We speculate that a more complex immune process is present in ICC due to its high malignancy.

Unsupervised clustering of macrophages (an important component of myeloid cells) and T cells were then performed with 11 clusters found, including four clusters for macrophages (Macro1, Macro2, Macro3, and Macro5) and seven clusters for T cells (CD4 CCR7, CD4 TIGIT, CD8 CCR6, CD8 GZMK, CD8 HSPA6, T cycling, and undefined) (Figure 4F,K and Figure S8). Usually, CD163 and CD68 are used to identify macrophages, which were highly expressed in cells identified in this study, especially in Macro1 and Macro2. Macrophage subtypes were more enriched in geneClusterB, consistent with previous results, which may affect tumor progression (Figure 4G,I). Meanwhile, patient bias existed, with patient H38 showing all four subtypes, patient H21 mainly showing Macro1 and Macro5, and patient C20 mainly showing Macro1 and Macro3, suggesting that the different microenvironments contribute to tumor heterogeneity (Figure 4H,J). Additionally, T cell subtypes were almost enriched in geneClusterB (Figure 4L,N). The diversity of T cells in ICC was higher than that in HCC (Figure 4 M,O). Due to the related genes, we designated a cluster of T cells as cycling cells, which were composed of CD4+ T cells, with high cytotoxicity (GZMA, NKG7, and GZMK) or transcription factor signals (TBX21, ZNF683, ZEB2, ID2, EOMES, HOPX, and TOX) (Figure S8B), implying a potential antitumor effect. In addition, another cluster of T cells that included CD8 GZMK cells with high cytotoxicity (GZMB, PRF1, GZMK, IFNG, GZMA, and NKG7) was classified, and it was consistent with previous reports,^23^ suggesting a strong explanation for the better prognosis in geneClusterB.

### 2.5 Features of transcriptional and posttranscriptional regulation based on the RH_Score model

**FIGURE 5:**
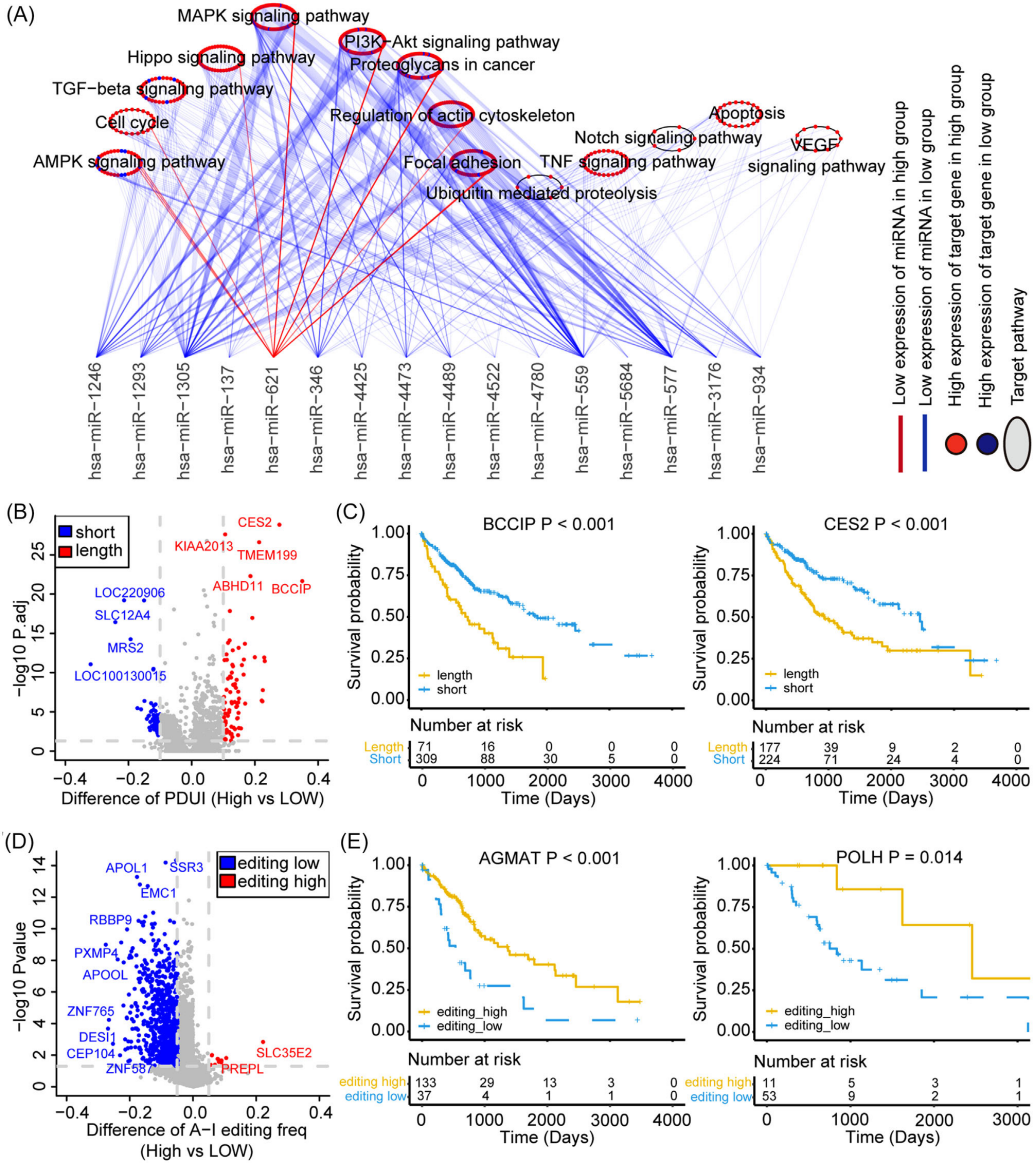
Features of transcriptional and posttranscriptional regulation based on the RH score. (A) Differences of miRNA‐targeted pathways in The Cancer Genome Atlas (TCGA)‐hepatobiliary malignancy (HBM) cohort based on different RH scores. Red line (low expression of miRNA in high score group); blue line (low expression of miRNA in low score group). Red dots (highly expressed targeted genes in high score group; blue dots (highly expressed targeted genes in low score group). The circle (signaling pathways). (B) Differences of the distal poly(A) site usage index (PDUI) of each gene in different RH score groups. Red (lengthening); blue (shortening); gray (no significant change). (C) Kaplan‐Meier curves displaying overall survival between PDUI lengthening (yellow) and shortening (blue) of BCCIP and CES2. The bottom of the chart shows the grouping of HBM. The difference was statistically significant when *p* < 0.05 in the log‐rank test. (D) Comparation of A‐I editing's frequency between the high and low RH score groups. Red (high A‐I editing); blue (low A‐I editing); gray (no significant change). (E) Kaplan‐Meier curves displaying overall survival between A‐I editing high (yellow) and A‐I editing low (blue) AGMAT and POLH. The bottom of the chart shows the grouping of HBM. The difference was statistically significant when *p* < 0.05 in the log‐rank test.

To further evaluate the role of the RH score in interpreting transcriptional and posttranscriptional events, we focused on the processes associated with RNA modification, including APA and A‐I editing. It has been reported that RNA modification patterns are related to different miRNA signatures with the effect of APA modification.^17^ Therefore, we explored differences in miRNA expression between high and low RH scores in the TCGA‐HBM cohort, screened 16 miRNAs with significant differences in expression, and performed enrichment analysis on their target gene signaling pathways (Table S11). Only miR‐621 showed low expression in the high‐score group with more oncogene enrichment, while other miRNAs had low expression in the low‐score group (Figure 5A), and the expression of these target genes varied greatly between the two groups. The miRNA‐targeted genes were significantly associated with the AMPK, cell cycle, TGF‐β, Hippo, MAPK, PI3K‐Akt, focal adhesion, TNF, Notch, apoptosis, and VEGF signaling pathways, which indicated that the RH score was closely related to the posttranscriptional regulation of miRNAs.

Furthermore, the APA and A‐I editing events of each gene in the TCGA‐HBM cohort were analyzed to find their posttranscriptional features (Tables S12 and S13). More genes with lengthening APA events were enriched in the high RH_Score group and were related to shorter survival (Figure 5B and Figure S9A). BCCIP was initially identified as an interacting protein of BRCA2 and CDKN1A (Cip1/waf1/p21).^24^ Abnormal regulation of BCCIP has been discovered in different cancers.^25^ BCCIP represents a paradoxical class of modulators for tumorigenesis as a suppressor for initiation but a requisite for progression.^26^ CES2 is a key enzyme activated by irinotecan, and the expression of CES2 in tumors may contribute to the variable response of solid tumors to irinotecan chemotherapy.^27^ BCCIP (Diff = 0.35, *p* < 0.001) and CES2 (Diff = 0.28, *p* < 0.001) exhibited significant lengthening, which showed shorter survival in patients with HBM (Figure 5C). In contrast to a previous study,^17^ we proposed the possibility that due to the prolongation of BCCIP and CES2 with the prolongation of the 3′UTR in turn in the high RH_Score group, more carcinogenic regulators could target the corresponding gene to intervene in the function of miRNA, leading to the activation of oncogenes and the progression of HBM.

Additionally, it has been found that A‐to‐I editing could alter the 3′UTR of mRNA and affect miRNA binding.^28^ The genes with a lower A‐to‐I editing rate were more enriched in the high RH_Score group and were related to poorer survival in HBM patients (Figure 5D and Figure S9B). AGMAT is upregulated in lung adenocarcinoma tissues, which promotes tumorigenesis by activating MAPK and PI3K/Akt cascades, and patients with high levels of AGMAT have a poor prognosis.^29^ DNA polymerase eta (POLH), as the Y‐family of DNA polymerases, mediates DNA translation synthesis and is associated with cisplatin resistance in cancer, which can be regulated by RNA modification.^30^ The low A‐I editing rates of AGMAT (Diff = −0.13; *p* < 0.001) and POLH (Diff = −0.117820911; *p* < 0.001) were related to poorer survival time in HBM patients (Figure 5E). The difference in the rate of A‐I editing of these genes between the high and low RH_Score groups may mediate the function of miRNA in the editing of the 3′UTR regions to affect the progression of HBM.

### 2.6 Potential therapeutic value of RNA modification patterns

**FIGURE 6:**
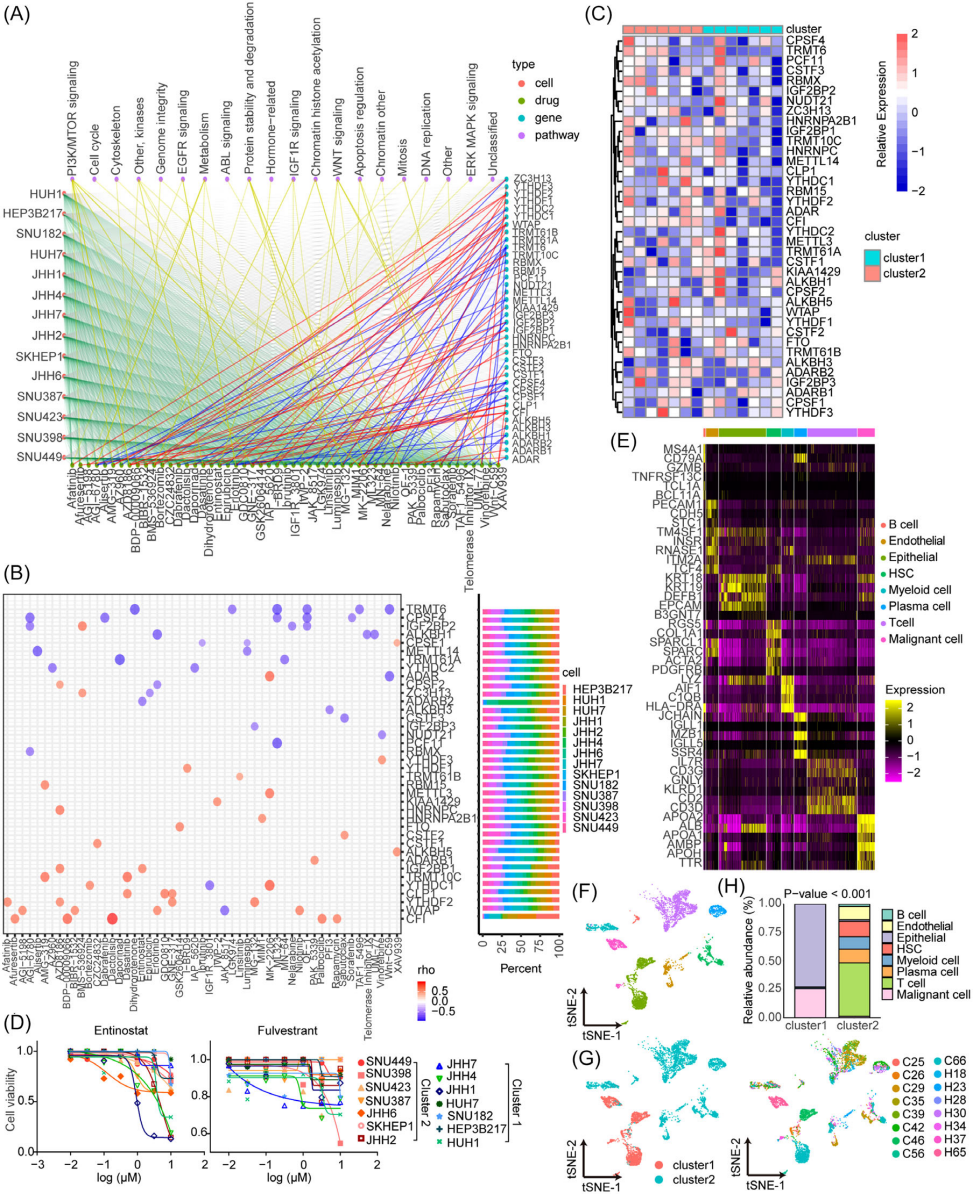
Relationship between RNA modification signatures and drug response and efficacy of immunotherapy. (A) Network diagram displaying the correlation among RNA modification signatures and drug sensitivity assessed using Spearman analysis. Red line: drug resistance (Rs > 0), blue line: drug sensitivity (Rs < 0), and yellow line: target pathways. (B) Disk chart displaying the correlation between RNA modification signatures and drug sensitivity assessed using Spearman analysis. Red: drug resistance (Rs > 0), blue: drug sensitivity (Rs < 0); bar chart (right panel): the expression of 41 RNA modification regulators. (C) Heatmap visualizing unsupervised clustering of 41 RNA modification regulators in 14 hepatobiliary malignancy (HBM) cell lines. The clusters of HBMs were used as sample annotations (top bar color label). Red (high expression); blue (low expression). Color bar: positive (red); negative (blue); Number: degree of correlation. (D) Effect of entinostat and fulvestrant on 14 HBM cell lines. The clusters are shown in the right annotation. (E) Heatmap displaying the marker genes’ expression in the indicated cell types. The color of the top bar marks the cluster corresponding to specific cell types. (F) t‐SNE plot displaying the annotation and color coding for cell types in patients with anti‐PD‐L1 treatment. (G) t‐SNE plot displaying the origin of cells by color and geneCluster (left) and a t‐SNE plot showing the origin of cells by color, patient origin (right). (H) Histogram indicating the proportion of cells in geneClusterA and geneClusterB. *p* < 0.05 was considered statistically significant.

To explore the effect of RNA modification patterns on drug response, we evaluated the relationship between 41 RNA modification regulators and drug response in 14 HBM cell lines, which could also be classified into cluster1 and cluster2 according to the previous classification method (Figure 6C and Table S14). A Spearman correlation analysis identified 77 pairs between drug sensitivity and 41 RNA modification regulators from the cancer drug sensitivity genomics (GDSC) database (Figure 6A,B). Of these pairs, 37 showed sensitivity, including daporinad (Rs = −0.886, *p* = 0.033), ML323 (Rs = −0.868, *p* < 0.001), dihydrorotenone (Rs = −0.864, *p* < 0.001), alisertib (Rs = −0.846, *p* < 0.001), erlotinib (Rs = −0.830, *p* < 0.001), Wnt‐C59 (Rs = −0.829, *p* < 0.001), AGI‐6780 (Rs = −0.811, *p* < 0.001), while the others showed resistance, including AZD8186 (Rs = −0.775, *p* = 0.003), AMG‐319 (Rs = −0.791, *p* < 0.001), MK‐2206 (Rs = −0.868, *p* < 0.001), dasatinib (Rs = −0.819, *Rs* = −0.001), and BDP < 0.001 (MK = −0.841, *p* < 0.001). In addition, drug sensitivity to RNA modification regulators was closely related to specific signaling pathways (Figure 6A‐B). In detail, we analyzed the effect of Entinostat and Fulvestrant on 14 HBM cell lines and found that Entinostat with drug resistance showed no difference in cluster1 and cluster2, while Fulvestrant with drug resistance exerted a more lethal effect in cluster1 HBM cell lines (Figure 6D). Taken together, these results suggest that RNA modification patterns are related to drug response.

Again, we implemented the l niche atlas with cell classification and marker gene identification using a single‐cell sequencing dataset (GSE125449) with the anti‐PD‐L1 treatment (Table S15). Consistent with the previous results, epithelial cells, and malignant cells were mainly enriched in geneClusterA, while myeloid cells, plasma cells, and T cells were still mostly enriched in geneClusterB (Figure 6E–H). Furthermore, unsupervised clustering of macrophages and T cells showed five clusters for macrophages (Macro1, Macro2, Macro3, Macro4, and Macro5) and nine clusters for T cells (CD4 CCR7, CD4 TIGIT, CD8 CCR6, CD8 GZMK, CD8 HSPA6, CD8 MT‐RNR2, T cycling and undefined) (Figures S10A–D and S11A–D). Similarly, the macrophage subtypes and T cell subtypes were more enriched in geneClusterB (Figures S10E and S11E). In addition, more diversity of T cells was observed in ICC than in HCC. Interestingly, macro4 was newly classified and mainly found in ICC, and macro5 was mainly found in HCC (Figures S10F and S11F). More importantly, there was a significant increase in the types of T cells in patients after anti‐PD‐L1 treatment, suggesting that the immune response in the TME was activated, leading to survival improvement in geneClusterB.

### 2.7 Prediction value of the RH score model for immunotherapy

**FIGURE 7:**
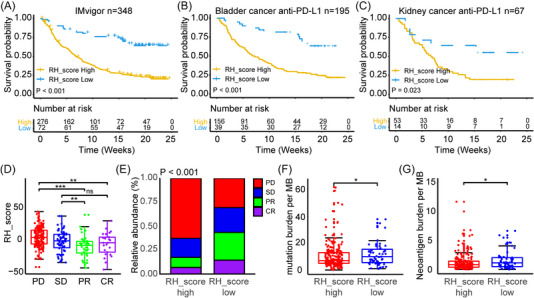
Prediction value of RH_Score model to immunotherapy. (A–C) Kaplan‐Meier curves show overall survival in the high RH_Score (yellow) and low RH_Score (blue) subgroups after the PD‐L1 blockade immunotherapy in the IMvigor210 (A), bladder cancer anti‐PD‐L1cohort (B), and kidney cancer anti‐PD‐L1cohort (C). The grouping of patients is shown at the bottom of the chart. *p* < 0.05 in the two‐sided log‐rank test was considered statistically significant. (D) The difference in the RH_Score between distinct clinical outcomes of anti‐PD‐L1 treatment in the IMvigor210 cohort. (E) The proportion of patients in the IMvigor210 cohort with different responses to PD‐L1 blockade immunotherapy. The fisher. Test was used to determine the statistical significance of the difference. SD, stable disease; PD, progressive disease; CR, complete response; PR, partial response. (F, G) Differences in tumor mutation burden (TMB) (F) and neoantigen burden (G) between high RH_Score (red) and low RH_Score (blue) groups in the IMvigor210 cohort. Wilcoxon test was used to assess the difference. The boxes indicate the median ± 1 quartile, with the whiskers extending from the hinge to the smallest or largest value within 1.5× IQR from the box boundaries. (Level of significance: ***, *p* < 0.001; **, *p* < 0.01; *, *p* < 0.05; ns, *p* > 0.05).

Given that the RH_Score appears to correlate with the TME (Figure S5D), we explored the effect of the RH_Score for predicting the response to immunotherapy based on the Imvigor210 cohort (Figure 7A–C). We found that high RH_Score patients in the anti‐PD‐L1 cohort, including the Imvigor210 cohort,^31^ bladder cancer cohort, and kidney cancer cohort, showed poorer prognoses. The Imvigor210 group showed four degrees of response to PD‐L1 blockers, such as complete response (CR), partial response (PR), disease stability (SD), and disease progression (PD). The RH_Score was lower in patients with PR and CR and highest in patients with PD (Figure 7D,E). Tumor mutation burden (TMB) has also been used to identify the effectiveness of immunotherapy.^32^ Therefore, the TMB and neoantigen burden were examined and were found to be decreased in the high RH score group compared with the low RH score group (Figure 7F,G), which may partially explain the survival disadvantage and resistance to immunotherapy in the high RH score group.

## 3 DISCUSSION

**FIGURE 8:**
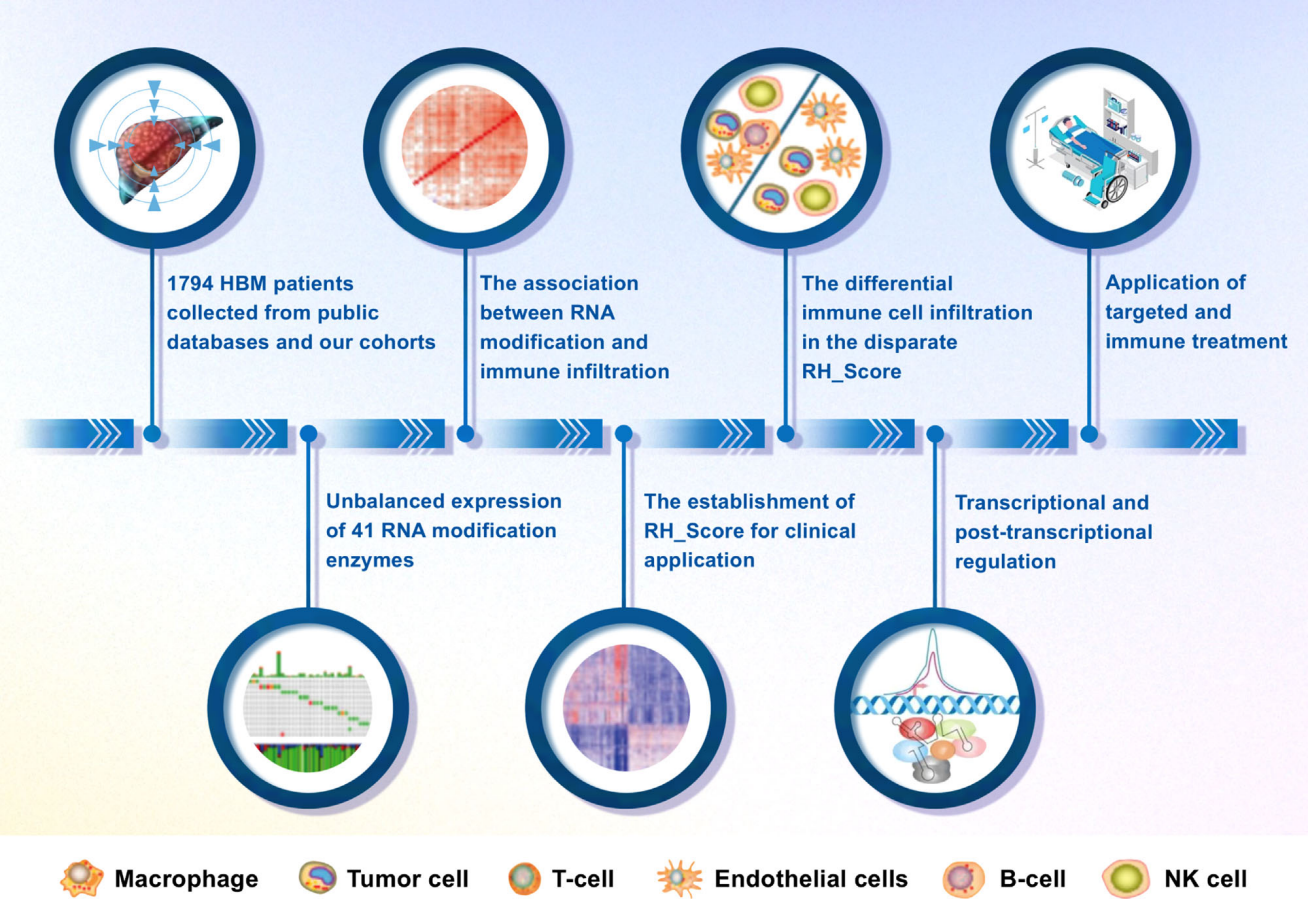
Graphical summary for comprehensive characterization of RNA modification in hepatobiliary malignancy (HBM).

As a rising research hotspot, the function of RNA modifications in certain cancers has been revealed. More than 100 different types of modifications have been shown to be present in RNA. Among them, four RNA modifications, namely, m^6^A, m^1^A, APA, and A‐I RNA editing, are the most common and the most studied types and they are primarily mediated by the “writer”, “reader” and “eraser” enzymes and constitute key mechanisms for epigenetic regulation of tumorigenesis.^6^ It was reported that the expression of METTL3, WATP, and KIAA1429 increased in HCC.^33^, ^34^ YTHDF2 silencing in human HCC cells provoked inflammation, vascular reconstruction, and metastatic progression.^35^ FTO was found to regulate the integrin signaling pathway, inflammation signaling pathway, epidermal growth factor receptor signaling pathway, angiogenesis, and pyrimidine metabolism pathway in ICC.^36^ In this study, we selected 41 RNA modification enzymes according to publicly available data and found that unbalanced expression of these regulators exists in HBM. Although the proportion of DNA mutations in HBM is not high, the 41 RNA modification regulators show significant differences at the transcription level and present extensive crosstalk between each other, implying certain representativeness in tumor and nontumor tissue, which is consistent with previous studies.^37^ Based on the expression imbalance, we divided the patients into different patterns (cluster1 and cluster2) and found that cluster1 was enriched in tumor‐related pathways, including mTOR, Notch, and the cell cycle, while cluster2 was enriched in metabolism‐related pathways, such as amino acid metabolism, lipid metabolism, sugar metabolism, and drug metabolism. These results suggest that RNA modification regulators affect the development and metabolism of HBM. Usually, high metabolism supports tumor initiation and progression.^38^, ^39^ Therefore, we speculated that low expression of RNA modification enzymes may induce the reprogramming of HBM metabolism to intervene in tumor progression and prognosis (Figure 8).

RNA modification enzymes also influenced immune infiltration of the TME, which played a significant role in tumor progression and prognosis. Researchers found that KIAA1429 (an m6A methyltransferase) resulted in less TME dendritic cell (DC) infiltration and decreased expression of MHC molecules.^12^ The loss of YTHDF1 in classic DCs can significantly enhance the cross‐presentation of tumor antigens and the cross‐activation of CD8^+^ T cells, which regulates durable neoantigen‐specific immunity.^40^ Additionally, RNA modification also maintains the immunosuppressive function of Treg cells.^41^ In this study, cluster1 showed that more immunosuppressive cells infiltrated, such as Tregs, M2 macrophages, and resting NK cells, whereas cluster2 exerted more immune‐promoting cells, such as CD8+ T cells, M1 macrophages, B cells, activated NK cells, and monocytes. This difference may be an important cause of the poorer prognosis in patients with HBM.

More importantly, a reliable predictive RH_Score model was established based on RNA modification enzymes to provide a brighter and more precise landscape for targeted drugs and immunotherapy applications. Many researchers have concentrated on this field. Chong et al.^42^ proposed the m^6^Sig score according to 23 m^6^A regulators, aiming to estimate prognosis and immunotherapy. The WM_score presented by Chen et al.^17^ based on 26 RNA modification “writers” showed correlations in colorectal cancer molecular subtypes, gene expression regulation, and therapy. However, these studies only included a single RNA modification method or partial participants in the RNA modification process, reflecting clinical survival and a lack of firmness in cellular and molecular analyses. Hence, we collected all the known “writer”, “reader,” and “eraser” enzymes and applied a comprehensive and convincing RH score model to identify immune infiltration types and survival prognosis in the field of HBM. In addition, the RH score could also predict the effectiveness of anti‐PD‐L1 in different clusters of patients, with patients in cluster B showing a better treatment effect (Figure 8).

From the perspective of RNA modification enzymes, TME analysis may be a ray of dawn for hepatobiliary tumor precision medicine. Although we enrolled 41 RNA‐modifying enzymes, other participants in the RNA modification process required inclusion. In addition, the predictive performance of the RH score still needs testing in other tumors and immunotherapy regimens, such as anti‐PD‐L1, anti‐cytotoxic T‐lymphocyte antigen‐4, therapeutic antibody, and adoptive T cell transfer.

## 4 CONCLUSIONS

In this study, we comprehensively analyzed RNA modifications in HBM that induce TME changes and transcriptional and posttranscriptional events, and the results may have potential guiding significance in prognosis prediction and treatment options.

## 5 MATERIALS AND METHODS

### 5.1 Public datasets data collection and preprocessing

The mRNA expression data, miRNA expression data, mutation data, CNV data, clinical information, and survival data of liver cancer and cholangiocarcinoma were collected from the TCGA database (https://gdc‐portal.nci.nih.gov/) and UCSC Xena data website (https://xenabrowser.net/datapages/). We acquired mRNA expression data of liver cancer samples from 5 GEO datasets (GSE76311, GSE136247, GSE112791, GSE55092, and GSE45436). Since these five datasets were gathered from different chip platforms with unclear clinical information and various confounding factors, we analyzed each data set individually to avoid false positive results.

The single‐cell dataset (GSE125449) was obtained from the NCBI Public Data Platform, which is single‐cell transcriptome profiling of HBM biospecimens from nine hepatocellular carcinoma and ten intrahepatic cholangiocarcinoma patients. Cell Ranger (https://10xgenomics.com) was used to retain cells with cell gene numbers and UMI numbers within the mean ± 2 SD and with a mitochondrial gene ratio of less than 10% as high‐quality cells and then ready for downstream analysis. Seurat^43^ (https://satijalab.org/seurat/) was used for cell filtration, standardization, clustering, cell subgroup classification, and marker gene screening.

In the aspect of cluster analysis, the top 16 PCs were chosen using a resolution parameter equal to 0.4 for the clustering of all cells. For the clustering of T lymphocytes, the top PCs were chosen using a resolution parameter equal to 0.4. For the clustering of myeloid cells, the top nine PCs were chosen using a resolution parameter equal to 0.4.

Detailed clinical immunotherapy data were downloaded from the IMvigor210 dataset^31^ (http://research‐pub.gene.com/IMvigor210CoreBiologies). Based on this dataset, we determined the relationship between the RH score and various cancer immunotherapies.

### 5.2 Degree of TME cell infiltration calculation

The relative abundance of 22 immune cell types in cancer samples was computed by the CIBERSORT, RRID:SCR_016955 algorithm (https://cibersort.stanford.edu/). For chip expression, data after quantile normalization were collected, and for RNA‐seq expression, nonnormalized data were selected. The number of permutation tests was set to 1000.

### 5.3 Establishment of RH score model

1. The R LIMMA, RRID:SCR_010943 package was used to analyze significantly different genes based on total mRNA expression values: log_2_ transformation FPKM of tumor samples from different clusters. FDR < 0.05 and |log_2_FC | > 1 were set as the thresholds for filter differences.
2. Single‐factor Cox regression analysis of the survival data of samples corresponding to each identified gene with a significant difference was conducted, and *p* < 0.05 was chosen as the screening threshold. Genes that met the filter criteria were used to calculate the RH score.
3. Construction of RH score: for each sample, RH score = ∑beta DEGs × Exp DEGs, where beta represents the independent prognostic coefficient obtained by single factor Cox regression analysis of this gene and Exp DEGs means the expression level of the differential gene.

### 5.4 Statistical analysis

The expression correlation coefficients of 41 RNA modification enzyme genes were calculated by distance‐based Spearman analysis. The Wilcoxon test was used to analyze the significance of the difference. To evaluate the effectiveness of the RH score model, we chose the ROC curve. The R survival package, survminer package, and Kaplan‐Meier curve were applied to evaluate the correlation between different classifications and information on actual survival prognosis, with log‐rank *p* < 0.05 considered significant. The “surv‐cutpoint” function could directly calculate the best cutoff value of the continuous independent variable, the RH score, that affected survival data when the univariate analysis was proceeding. This cutoff value divides samples into the RH score high group and the RH score low group. Multivariate Cox regression analysis included factors such as high/low RH score, sex, age, and clinical stage to evaluate the relationship between these factors and survival and then demonstrated the reliability of high/low RH score as an independent factor affecting survival.

## AUTHOR CONTRIBUTIONS

Feng Qi and Jia Li carried out the conceptualization. Biwei Yang, Bin Zhou, Wenxing Qin, and Feng Qi participated in the clinical experimental analyses and statistical evaluations. Feng Qi, Jia Li, and ZRQ participated in the statistical and bioinformatics analyses. Feng Qi, Jia Li, and ZRQ drafted the manuscript. FQ and JZ reviewed and edited the manuscript. All authors have read and approved the final manuscript.

## CONFLICT OF INTEREST STATEMENT

The authors declare no conflict of interest.

## ETHICS STATEMENT

The patient cohort we used was a publicly available dataset that was collected with patients’ informed consent. The study protocol was approved by the Ethics Committee of Fudan University Shanghai Cancer Center (Shanghai, China, No. 050432‐4‐2108).

## Supporting information



Supporting Information

Supporting Information

## Data Availability

The Gene Expression Omnibus (GEO) and The Cancer Genome Atlas (TCGA) are open databases available at https://www.ncbi.nlm.nih.gov/geo/ and https://www.cancer.gov/. Accession numbers of GEO are shown in Table S2. The transcriptomics data and raw sequencing data of the 100 samples in a validated cohort have been uploaded to the National Center for Biotechnology Information (NCBI) Gene Expression Omnibus (https://www.ncbi.nlm.nih.gov/geo/), with an accession number of GSE222334.
