# Supplementary material for: Modeling cross‐talk of RNA modification enzymes reveals tumor microenvironment‐associated clinical significance and immunotherapy prediction in hepatobiliary malignancy
Source: MedComm (2020). 2023 Apr 18;4(3):e256. doi: 10.1002/mco2.256 (PMC10113697; doi:10.1002/mco2.256)
Supplement: Supplementary file 1 — Supporting Information [file MCO2-4-e256-s002.docx]

**Supplementary information for the manuscript**

**Modeling cross-talk of RNA modification enzymes reveals tumor microenvironment associated clinical significance and immunotherapy prediction in hepatobiliary malignancy**

Feng Qi^1,2#*^, Jia Li^3#^, Zhuoran Qi^3#^, Bin Zhou^4#^, Biwei Yang^3^, Jun Zhang^2*^ and Wenxing Qin^1*^

1. Phase I Clinical Trial Center, Fudan University Shanghai Cancer Center, No. 270, Dong’an Road, Shanghai 200032, China; Department of Oncology, Shanghai Medical College, Fudan University, Shanghai, 200032, China;

2. Department of Oncology, Ruijin Hospital, Shanghai Jiao Tong University School of Medicine, Shanghai 200000, China;

3. Liver Cancer Institute, Zhongshan Hospital, Fudan University, Shanghai 200032, China;

4. Department of Hepatic Surgery VI, Eastern Hepatobiliary Surgery Hospital, Second Military Medical University, Shanghai 200433, China

***Corresponding authors**

Prof. Wenxing Qin, Department of Oncology, Second Affiliated Hospital of Naval Medical University, Shanghai 200003, China; Email: qinwenxingqwx@163.com

Prof. Jun Zhang, Department of Oncology, Ruijin Hospital, Shanghai Jiao Tong University School of Medicine, Shanghai 200000, China, E-mail: junzhang10977@sjtu.edu.cn

Dr. Feng Qi, Department of Oncology, Ruijin Hospital, Shanghai Jiao Tong University School of Medicine, Shanghai 200000, China, E-mail: [gluckqi@163.com](mailto:junzhang10977@sjtu.edu.cn).

#Feng Qi, Jia Li, Zhuoran Qi and Bin Zhou contributed equally to this research work.

**Supplementary Materials and methods**

***Cluster analysis focusing on the expression consistency of 41 RNA modification enzyme genes***

Unsupervised clustering is a method that can identify the number and members of possible clusters in a dataset (gene expression). R Consensus-Clusterplus package was used to perform consistent cluster analyses on the expression of 41 RNA modification enzyme genes consist of 7 m^6^A modification “writer” enzymes (METTL3, METTL14,WTAP, RBM15, RBM15B, ZC3H13 and KIAA1429), 4 m^1^A modification “writer” enzymes (TRMT61A, TRMT61B,TRMT10C and TRMT6), 12 APA modification “writer” enzymes (CPSF1-4, CSTF1/2/3, PCF11, CFI, CLP1, NUDT21 and PABPN1), 3 A-I modification “writer” enzymes (ADAR, ADARB1 and ADARB2), 3 m6A modification “eraser” enzymes (FTO,ALKBH5 and ALKBH), 2 m^1^A modification “eraser” enzymes (ALKBH1 and ALKBH3), 11 m^6^A modification “reader” enzymes (YTHDC1/2, YTHDF1/2/3, IGF2BP1/2/3, HNRNPA2B1, HNRNPC/G and eIF3), using the distance-based Kmean method, setting reps (the number of subsets) to 1000, and setting the number of clusters to 2 ^1^.

***Gene set variation analysis (GSVA) and functional enrichment analysis***

Gene set variation analysis (GSVA) is an algorithm of SeqGSEA, RRID:SCR_005724 (http://www.gsea-msigdb.org/) ^2^. Starting from the gene expression level of each sample and the information of the multiple function set gene set (c2.cp.kegg. v7.4, h.all.v7.4), the samples are unsupervised classified according to the changes of each function. The enrichment score of each sample was calculated, then the R LIMMA, RRID:SCR_010943 package was used to analyze the differences, and the R package GSVA ^3^ was used to complete the analysis.

For the data processing of the enrichment of GO and KEGG, RRID:SCR_012773 functions of differential genes, the R GSEABase package and clusterProfiler, RRID:SCR_016884 package ^4^ were applied. The false discovery rate (FDR) was obtained following the method of Benjamini and Hochberg adjustment. To control the largely increased false positive probability in multiple comparisons, a FDR < 0.05 was set as the filter condition.

***Study on the correlation between RH score and miRNA***

The miRNA data of tumor samples came from the TCGA database. Differences in miRNAs and mRNAs between the RH score high group and the RH score low group were analyzed by the Wilcoxon test with the Benjamini and Hochberg test used to adjust and identify differences with FDR < 0.05 (statistically significant). The target gene prediction of miRNA adopted the R multiMiR package (http://multimir.ucdenver.edu/) ^5^. KEGG functional enrichment analysis was performed on the selected miRNA-mRNA.

***RNA modification enzymes and GDSC drug sensitivity***

From Genomics of Drug Sensitivity in Cancer (GDSC: http://www.cancerrxgene.org/) ^6^, the IC50 values (the half maximal inhibitory concentration), target genes and pathways of 175 drugs in 14 liver cancer cell lines were obtained. The RNA-seq expression of 41 RNA modification enzymes in liver cancer cell lines was acquired from Cancer Cell Line Encyclopedia (CCLE: https://portals.broadinstitute.org/ccle). The relationship between each drug and the expression of 41 RNA modification enzymes was computed, and the results was statistically significant if the following two conditions were satisfied: absolute value of the correlation coefficient was greater than 0.6 and *p* < 0.05. A positive correlation represented drug resistance, and a negative correlation meant drug sensitivity.

According to the expression consistency of 41 RNA modification enzymes, 14 liver cancer cell lines were divided into 2 types. The relationships between the AUC of Entinostat and Fulvestrant in different cell lines and different concentrations were analyzed.

***Research on the correlation between RH score and APA***

The Cancer 3′ UTR Atlas (TC3A, http://tc3a.org) used the mature DaPars algorithm (https://github.com/ZhengXia/DaPars) to identify the alternative proximal polyA site and then calculated the Percentage of Distal polyA site Usage Index (PDUI) for each transcript. We obtained the PDUI of each gene in TCGA liver cancer and cholangiocarcinoma samples through Synapse, RRID:SCR_006307: syn24982198 and identified the genes that prolonged or shortened the PDUI of the RH score high group and RH score low group. This identification method used the *t* test. Benjamini- and Hochberg-adjusted FDR and FDR < 0.05 were considered statistically significant; at the same time, the difference in PDUI > 0.1. Furthermore, single-factor Cox regression was performed to screen out PDUI genes related to survival prognosis.

***Study on the correlation between RH score and A to I***

We obtained the A-to-I RNA editing profile data of liver cancer from Synapse: syn2374375^7^, 3'UTR gene region and A-to-I RNA editing level value (0-100%) of Alu elements between the RH score high group and RH score low group were compared. The *t* test was applied, and *p* < 0.05 meant statistically significant, together with a threshold (editing level difference degree > 5%) to screen out meaningful genes. Then, univariate Cox regression was used to identify genes related to survival prognosis.

**Supplementary Figures**


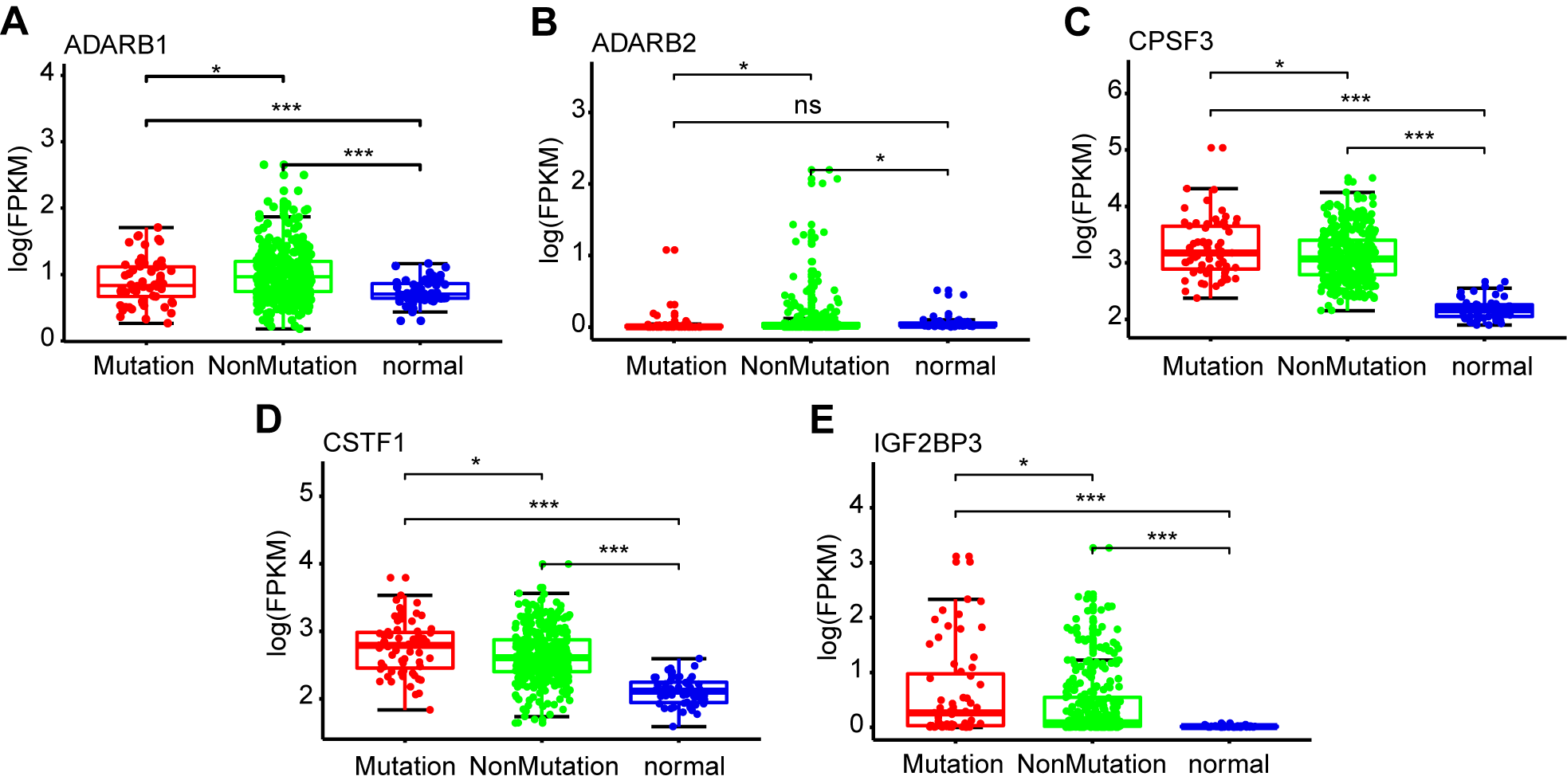


**Figure S1. Analysis of mutation in TCGA-HBM.** (A-E) Box plots show the expression distribution of several RNA modification regulators among mutation (red), non-mutation (green) and normal (blue) group. ADARB1 (A), ADARB2 (B), CPSF3 (C), CSTF1 (D), IGF2BP3 (E). The boxes indicate the median ± 1 quartile, with the whiskers extending from the hinge to the smallest or largest value within 1.5° ×IQR from the box boundaries. (Level of significance: ***, *P* < 0.001; **, *P* < 0.01; *, *P* < 0.05; ns, *P* > 0.05).


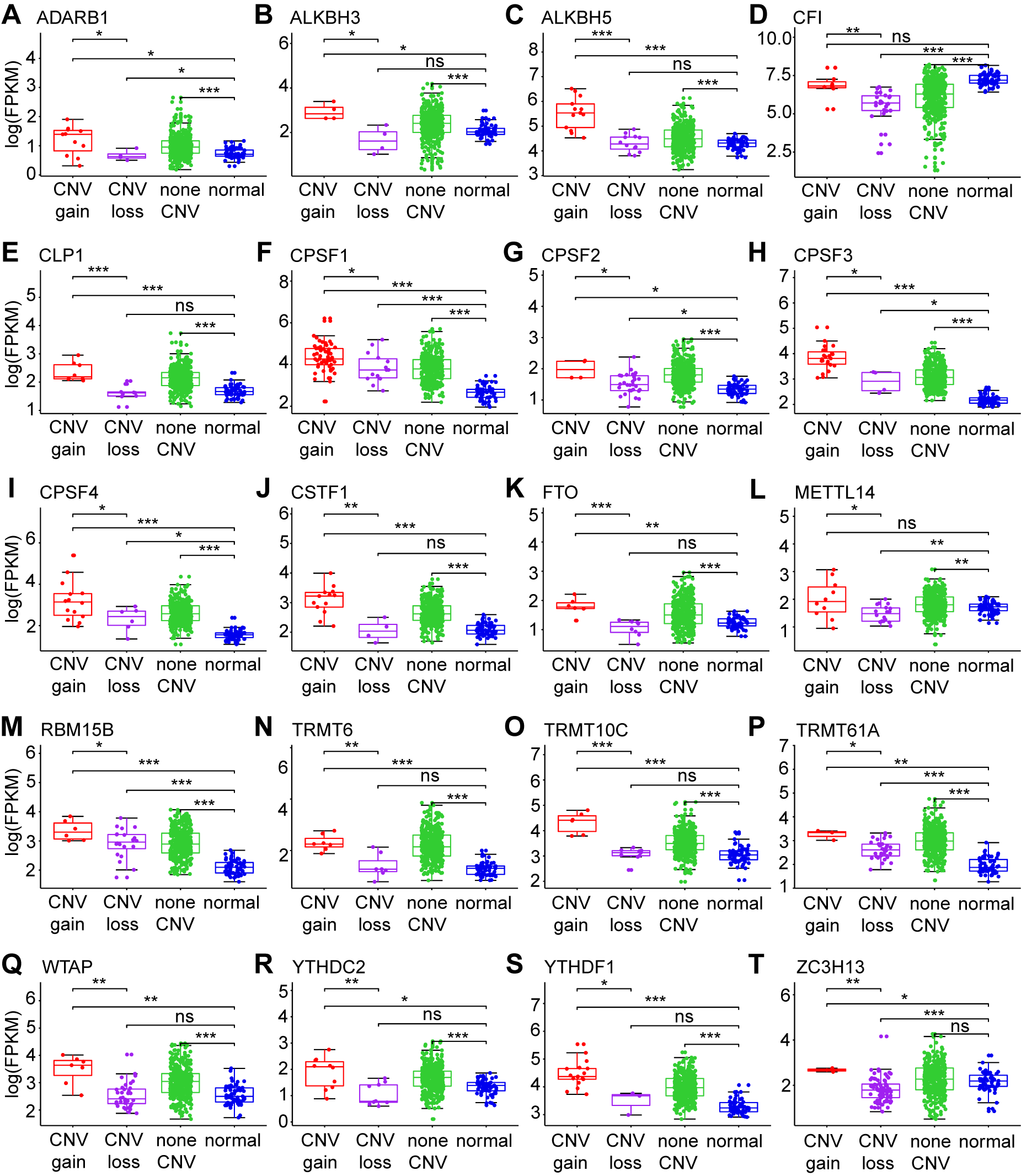


**Figure S2.** **Analysis of CNV** **in** **TCGA-HBM. (A-T) The** **expression of RNA modification regulators among CNV groups in HBM.** The sample size for each group based on the CNV alteration (CNV_gain/ CNV_loss/ none_CNV / normal). ADARB1 (A), ALKBH3 (B), ALKBH5 (C), CFI (D), CLP1 (E), CPSF1 (F), CPSF2 (G), CPSF3 (H), CPSF4 (I), CSTF1 (J), FTO (K), METTL14 (L), RBM15B (M), TRMT6 (N), TRMT10C (O), TRMT61A (P), WTAP (Q), YTHDC2 (R), YTHDF1 (S), ZC3H13 (T). Wilcoxon test was used to assess the difference. The boxes indicate the median ± 1 quartile, with the whiskers extending from the hinge to the smallest or largest value within 1.5× IQR from the box boundaries. (Level of significance: ***, *P* < 0.001; **, *P* < 0.01; *, *P* < 0.05; ns, *P* > 0.05).


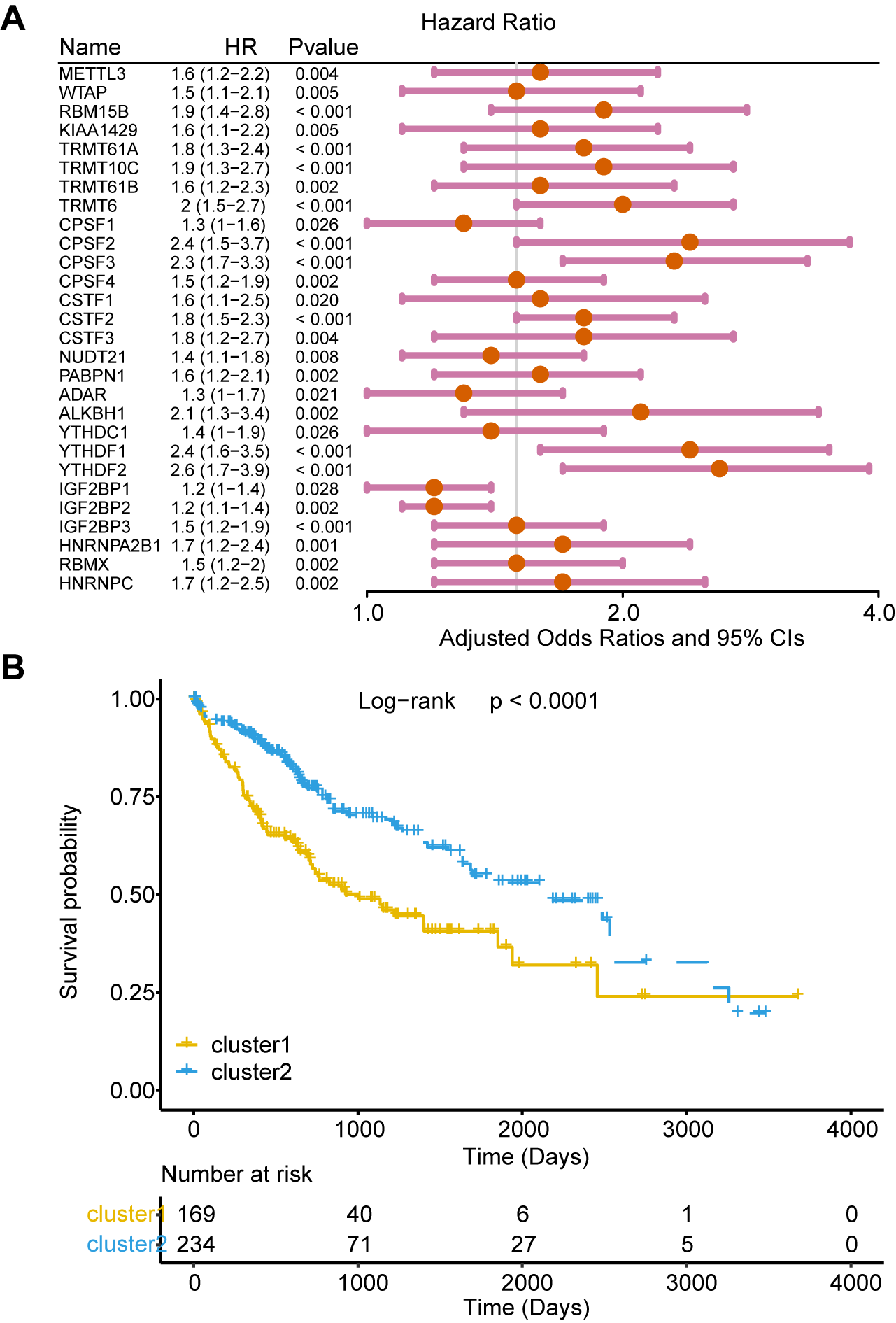


**Figure S3****. Biological characteristics of RNA modification** **regulators.** (A) Association of gene expression for 41 RNA modification regulators with patient overall survival times based on Univariate Cox regression analysis in TCGA-HBM. (B) Kaplan-Meier curves show overall survival of patients in cluster1 or cluster2 based on the expression of RNA modification regulators in the HBM cohort. p < 0.05 in the two-sided log-rank test was considered statistically significant.


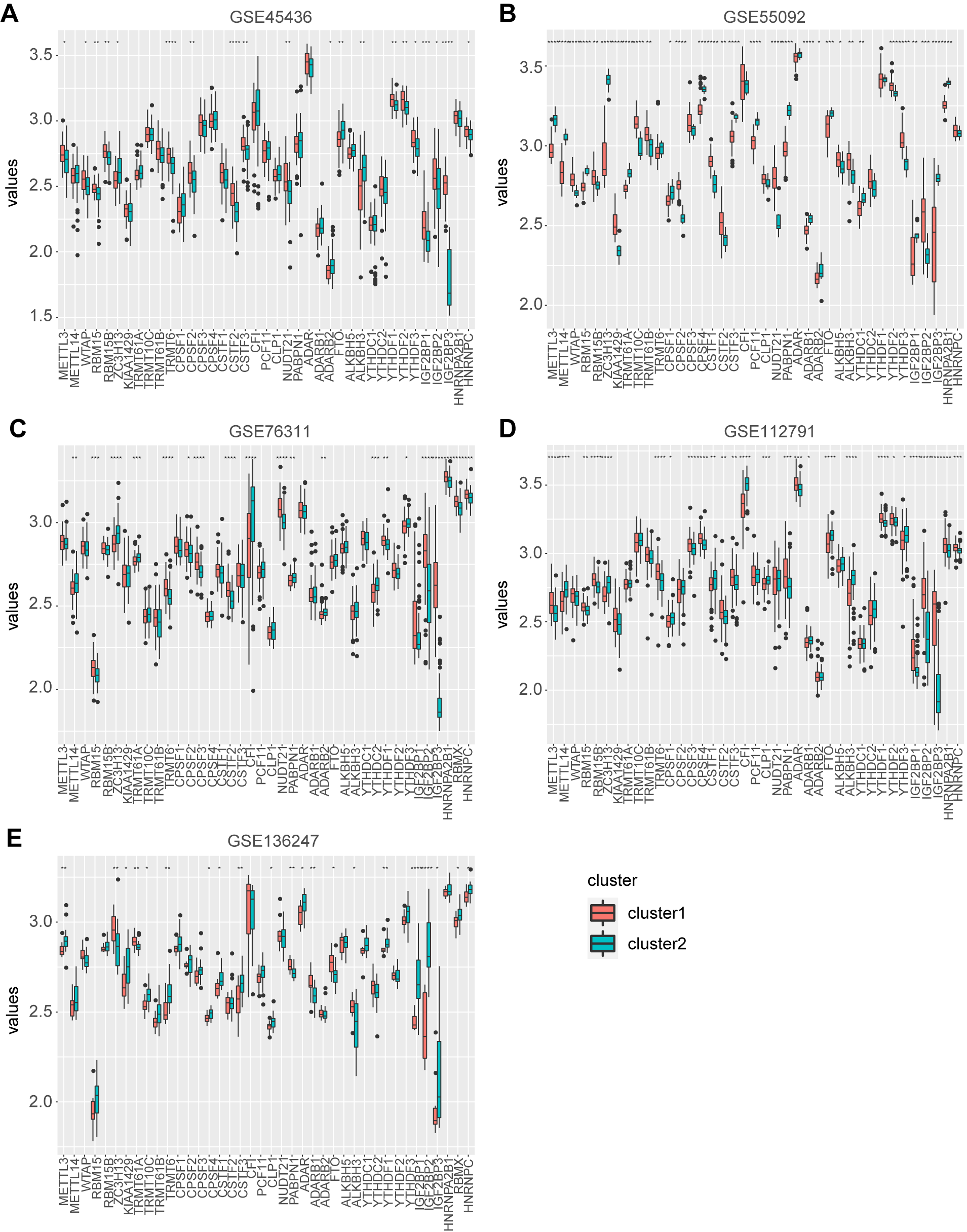


**Figure S4. Expression characteristics of RNA modification regulators in 5 GEO datasets.** (A-E) Box plots show differential expression of 41 RNA modification regulators between cluster1 or cluster2 in 5 GEO datasets. GSE45436 (A), GSE55092 (B), GSE76311 (C), GSE112791 (D), GSE136247 (E).


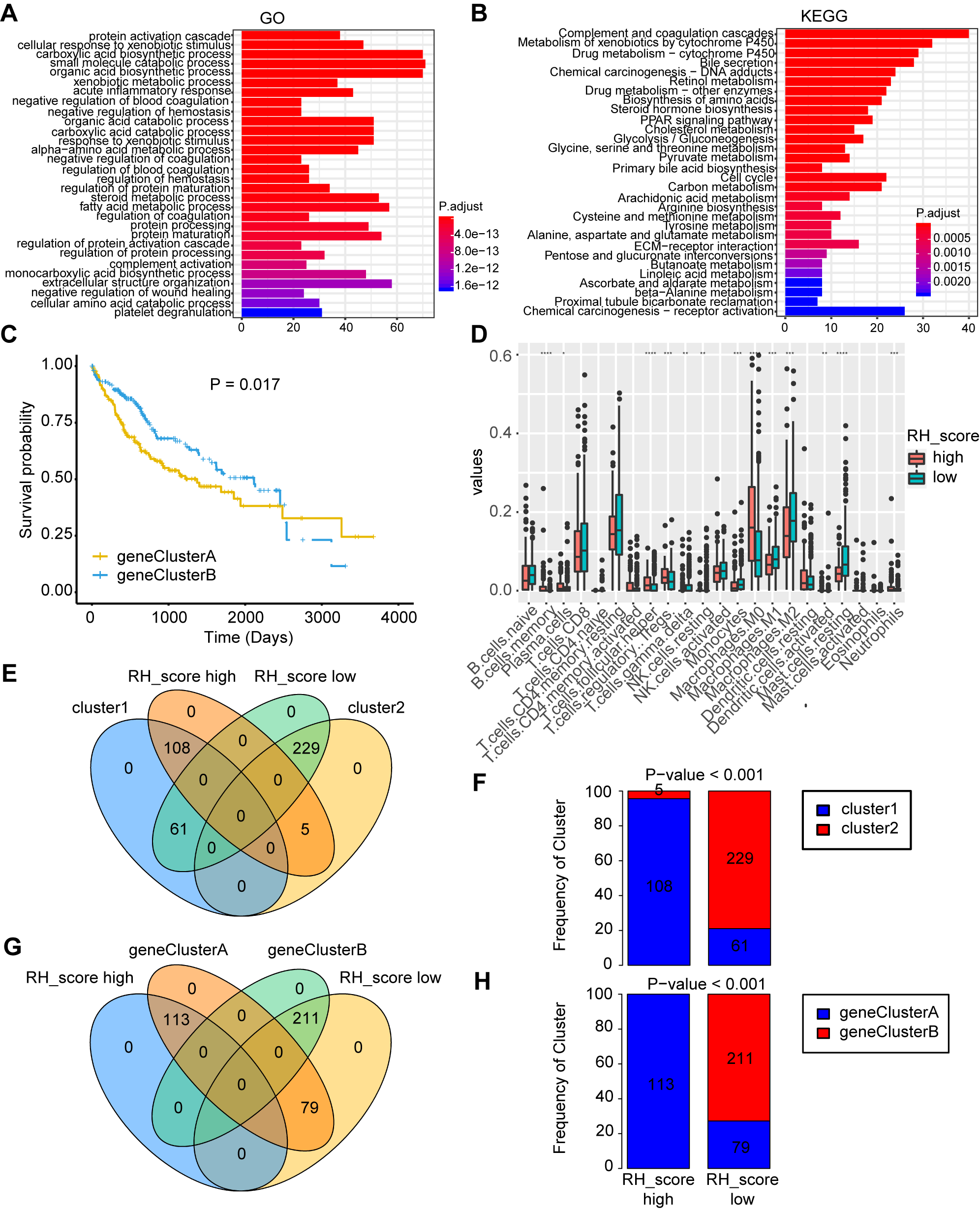


**Figure S5. Enrichment analysis of differentially expressed genes and the relationship between survival and the RH_Score.** (A-B) GO (A) and KEGG (B) enrichment analysis of the 924 DEGs. The x-axis indicates gene counts within each term. The brightness of the column color represents the statistical significance of enrichment. (C) Kaplan-Meier curves comparing overall survival between two DEG clusters, geneClusterA (yellow) and geneClusterB (blue), in TCGA. The grouping of HBM samples is shown under the Kaplan-Meier plot. p < 0.05 in the two-sided log-rank test was considered statistically significant. (D) Box plots show the differences in TME infiltration between high and low RH_Score groups. Orange, high score; Green, low score. (E-F). Overlap (E) and frequency (F) of classifiers of high/low RH_Score and cluster1/2 in HBM (G-H). Overlap (G) and frequency (H) of classifiers of high/low RH_Score and geneClusterA/B in HBM. The Fisher test was used to determine the statistical significance of the difference.


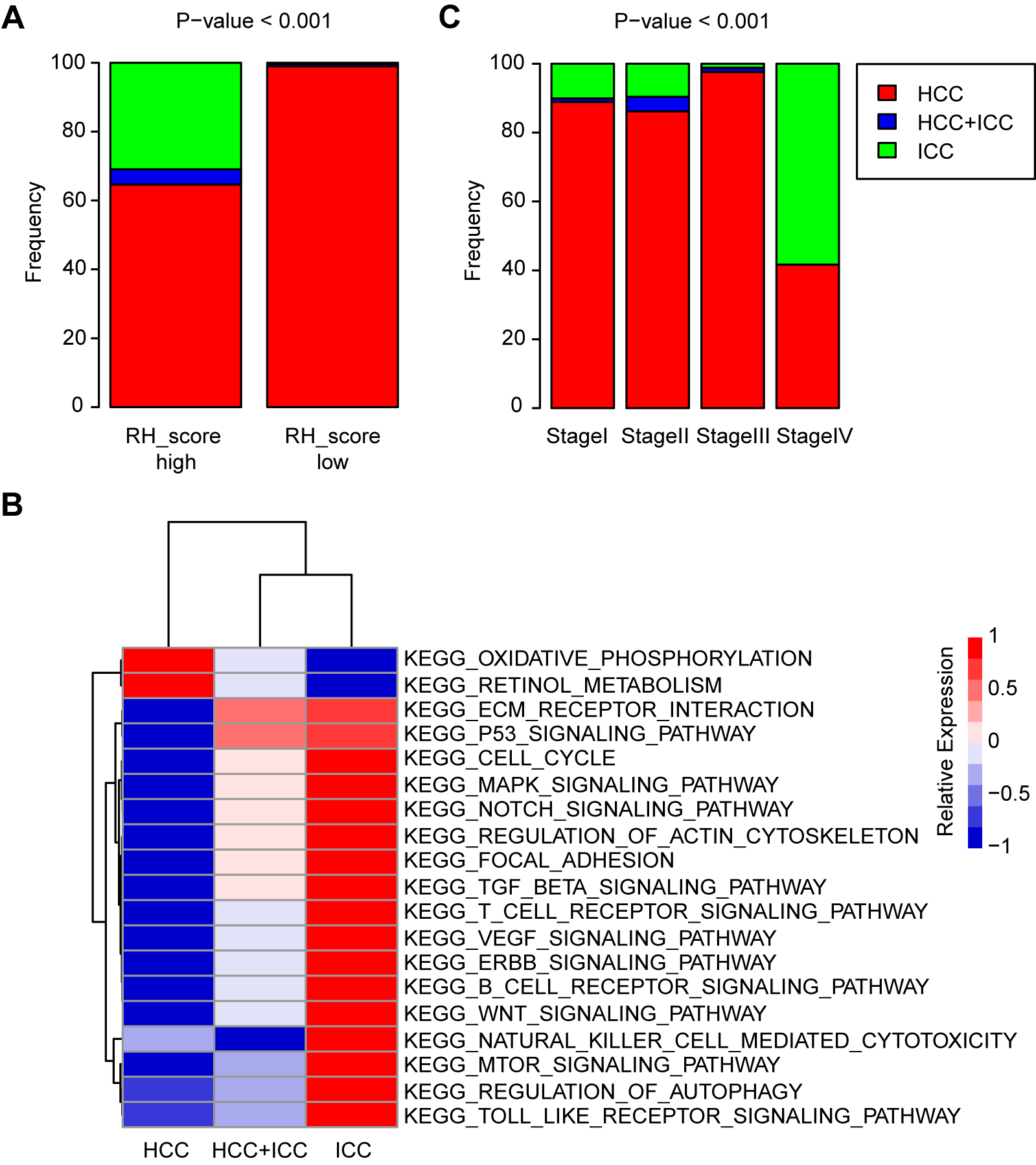


**Figure S6. Relationship between the RH_Score and the** **pathological subtypes and TNM stages of HBM.** (A) Distribution of HBM pathological subtypes within high and low RH_Score groups in TCGA. (B) Enrichment in signaling pathways in HBM pathological subtypes in TCGA. The number besides color bar is correlation coefficient. (C) Distribution of TNM stage within HBM pathological subtypes in TCGA. Statistical significance (p < 0.05) was calculated using the fisher. Test.


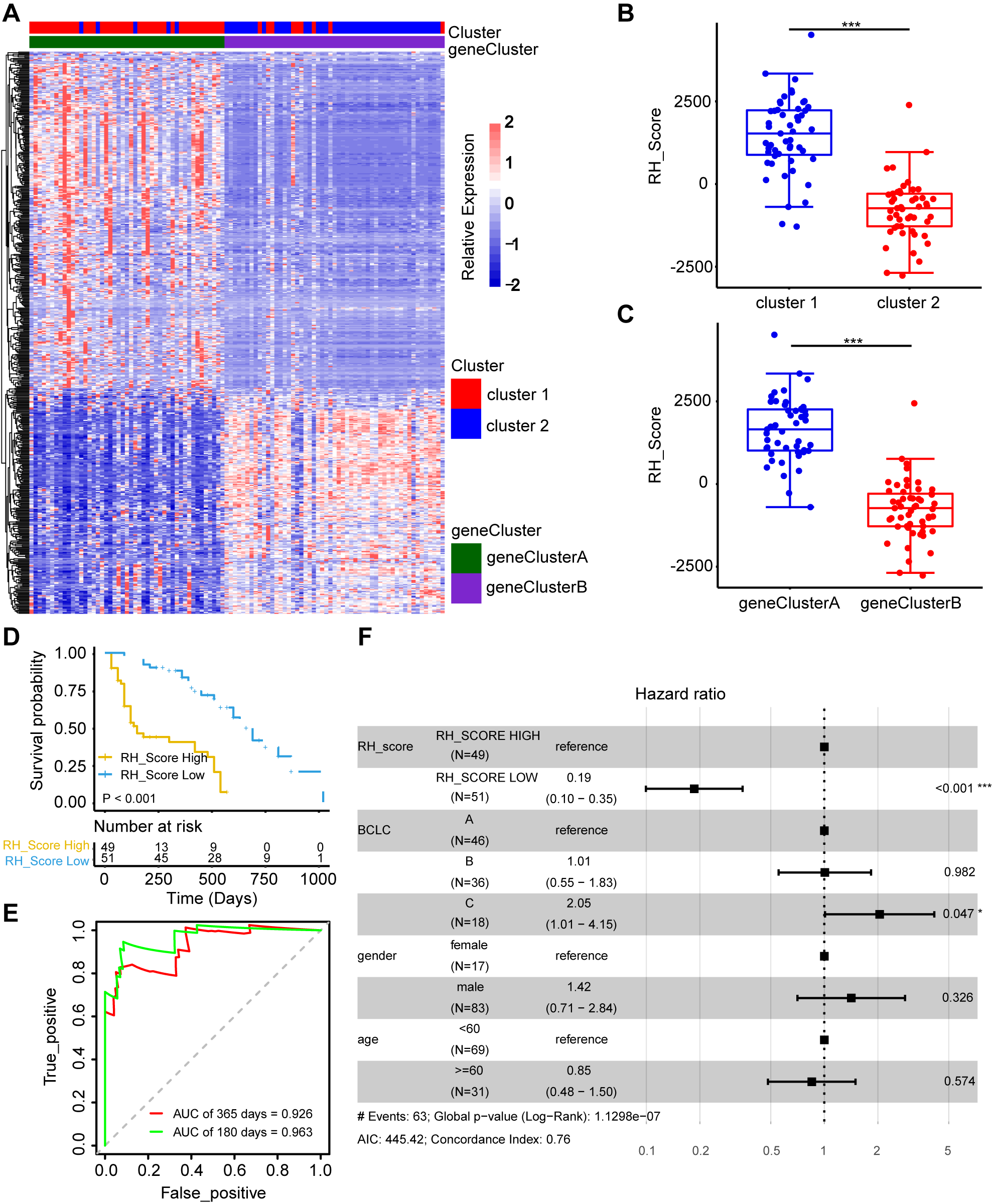


**Figure S7. Establishment of** **RNA modification signatures and RH_Score with clinical characteristics in validate cohorts.** (A) Unsupervised clustering of the RNA modified phenotype-related genes. The clusters of HBM were used as sample annotations. Red (high expression); blue (low expression). The number besides color bar is correlation coefficient. (B-C) Differences of RH score between RNA modified clusters (B) and gene clusters (C) in the validate cohort. Wilcoxon test was used, and P < 0.05 was considered statistically significant. (D) Kaplan-Meier curve displaying overall survival in RH_Score-high (yellow) and RH_Score-low (blue) validate cohort. The difference was statistically significant when P < 0.05 in log-rank test. (F) Predictive value of the RH score in patients from validate cohort (AUC: 0.963 and 0.926; 180- and 365-day overall survival).


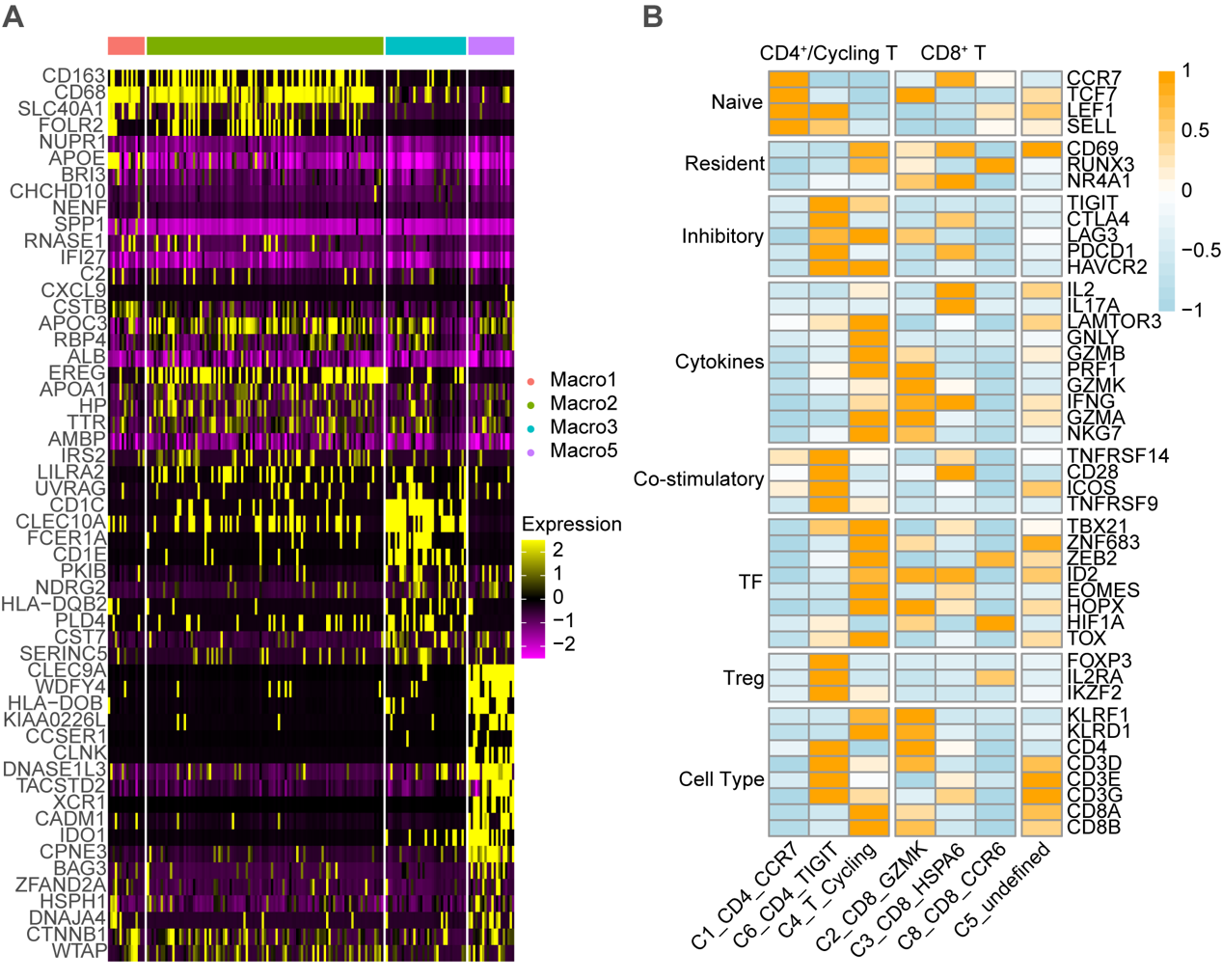


**Figure S8. The subtypes of Myeloid-derived cells and T cells in HBM patients without anti-PD-L1 treatment.** (A) Heatmap showing the expression of marker genes in the macrophage cell types. The top bar colors label the clusters corresponding to specific cell types. The number besides color bar is correlation coefficient. (B) Heatmap indicating the expression of selected gene sets in T subtypes, including naive, resident, inhibitory, cytokines, co-stimulatory, transcriptional factors (TF), and cell type. The number besides color bar is correlation coefficient.

**
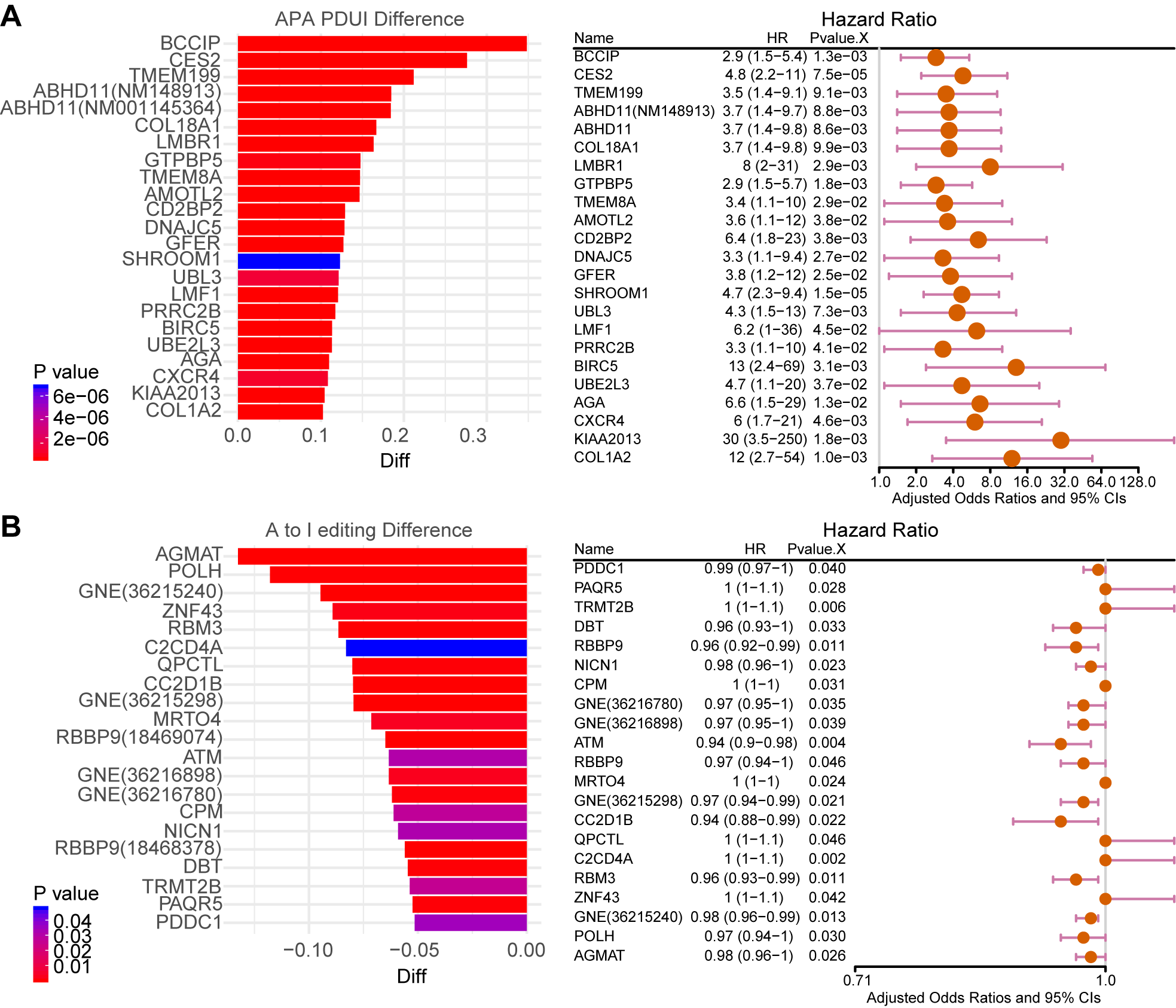
Figure S9. The length of APA PDUI gene and A-I editing gene affect the survival prognosis of HBM.** (A-B) The bar graphs show the difference between high and low RH_Score groups in PDUI (A) and A-I editing (B). The forest plots show univariate Cox regression analyses for PDUI differential genes (A) and A-I editing differential genes (B) between high and low RH_Score groups.


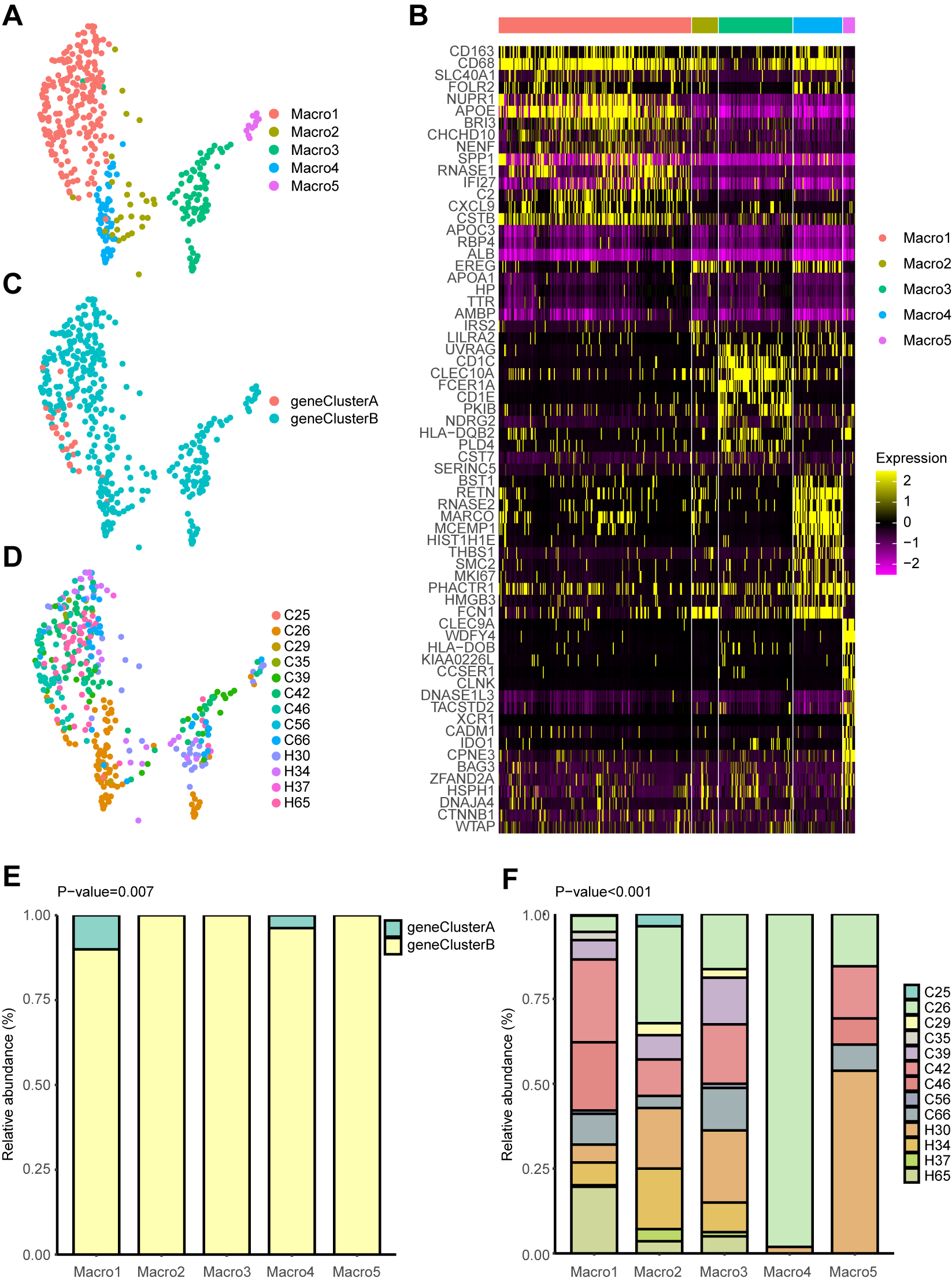


**Figure S10. Myeloid-derived cell main components in HBM patients with anti-PD-L1 treatment.** (A) T-distributed stochastic neighbor embedding (t-SNE) plot, showing the annotation and color codes for macrophage types in the HBM ecosystem. (B) Heatmap showing the expression of marker genes in the indicated cell types. The top bar colors label the clusters corresponding to specific cell types. The number besides color bar is correlation coefficient. (C) The t-SNE plot, showing cell origins by color, geneClusters (right panel). (D) The t-SNE plot, showing cell origins by color, patient origin (right panel). (E) Histogram indicating the proportion of cells in geneClusterA and geneClusterB. (F) Histogram indicating the proportion of cells in tumor tissue of each analyzed patient. p < 0.05 was considered statistically significant.


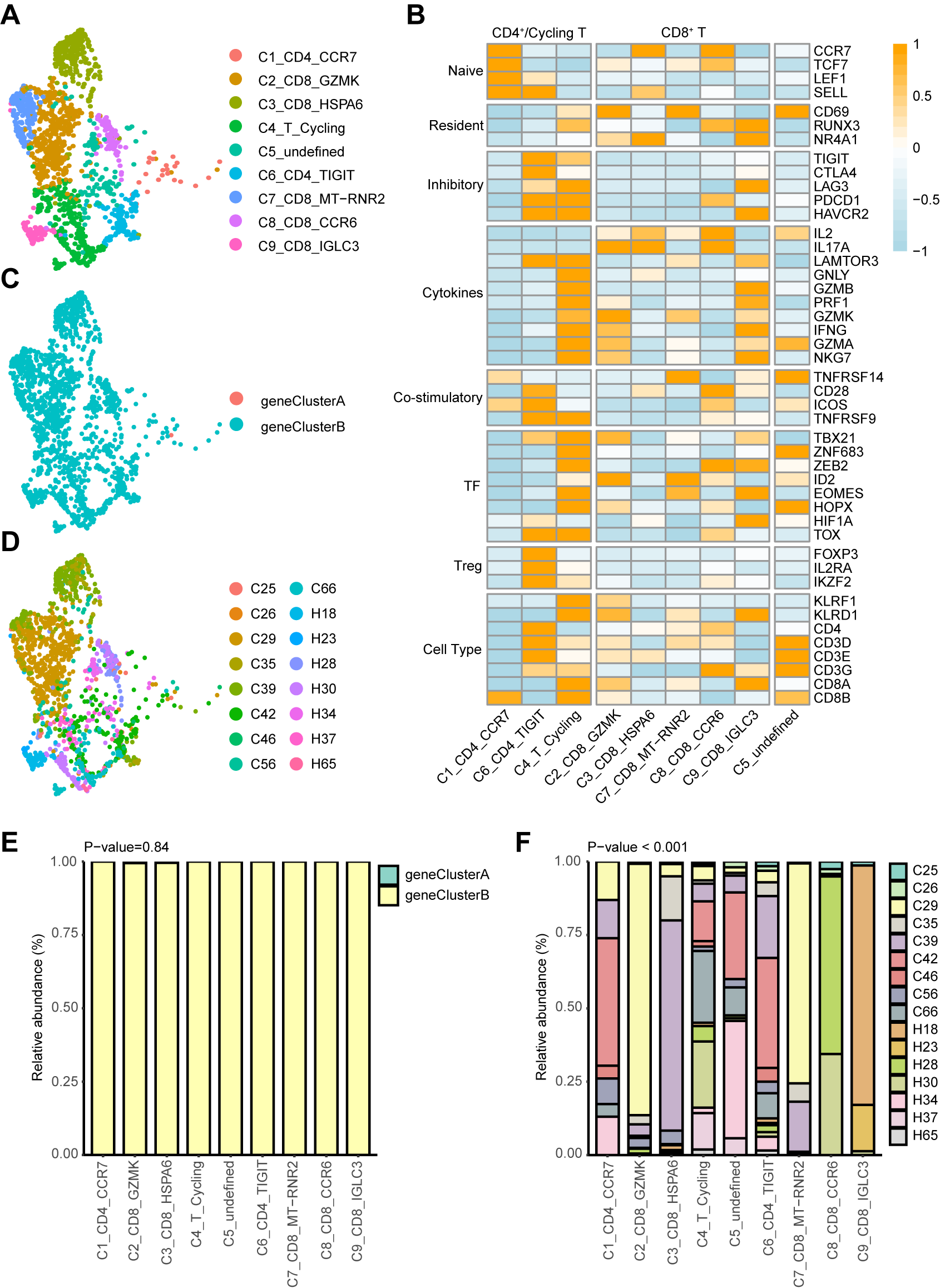


**Figure S11. The subtypes of T cells in HBM patients with anti-PD-L1 treatment.** (A) T-distributed stochastic neighbor embedding (t-SNE) plot, showing the annotation and color codes for T cell types in the HBM ecosystem. (B) Heatmap indicating the expression of selected gene sets in T subtypes, including naive, resident, inhibitory, cytokines, co-stimulatory, transcriptional factors (TF), and cell type. The number besides color bar is correlation coefficient. (C) The t-SNE plot, showing cell origins by color, geneClusters (right panel). (D) The t-SNE plot, showing cell origins by color, patient origin (right panel). (E) Histogram indicating the proportion of cells in geneClusterA and geneClusterB. (F) Histogram indicating the proportion of cells in tumor tissue of each analyzed patient. p < 0.05 was considered statistically significant

**References**

1. Wilkerson MD, Hayes DN. ConsensusClusterPlus: a class discovery tool with confidence assessments and item tracking. *Bioinformatics*. 2010;26(12):1572–3.

2. Subramanian A, Tamayo P, Mootha VK, et al. Gene set enrichment analysis: a knowledge-based approach for interpreting genome-wide expression profiles. *Proc Natl Acad Sci U S A*. 2005;102(43):15545–50.

3. Hänzelmann S, Castelo R, Guinney J. GSVA: gene set variation analysis for microarray and RNA-seq data. *BMC Bioinformatics*. 2013;14:7.

4. Yu G, Wang LG, Han Y, He QY. clusterProfiler: an R package for comparing biological themes among gene clusters. *Omics*. 2012;16(5):284–7.

5. Ru Y, Kechris KJ, Tabakoff B, et al. The multiMiR R package and database: integration of microRNA-target interactions along with their disease and drug associations. *Nucleic Acids Res*. 2014;42(17):e133.

6. Yang W, Soares J, Greninger P, et al. Genomics of Drug Sensitivity in Cancer (GDSC): a resource for therapeutic biomarker discovery in cancer cells. *Nucleic Acids Res*. 2013;41(Database issue):D955–61.

7. Han L, Diao L, Yu S, et al. The genomic landscape and clinical relevance of A-to-I RNA editing in human cancers. *Cancer Cell*. 2015;28(4):515–528.
